# Tank binding kinase 1 is a centrosome-associated kinase necessary for microtubule dynamics and mitosis

**DOI:** 10.1038/ncomms10072

**Published:** 2015-12-10

**Authors:** Smitha Pillai, Jonathan Nguyen, Joseph Johnson, Eric Haura, Domenico Coppola, Srikumar Chellappan

**Affiliations:** 1 Department of Tumor Biology, H. Lee Moffitt Cancer Center and Research Institute, 12902 Magnolia Drive, Tampa, Florida 33612, USA; 2 Department of Thoracic Oncology, H. Lee Moffitt Cancer Center and Research Institute, 12902 Magnolia Drive, Tampa, Florida 33612, USA; 3 Department of Anatomic Pathology, H. Lee Moffitt Cancer Center and Research Institute, 12902 Magnolia Drive, Tampa, Florida 33612, USA

## Abstract

TANK Binding Kinase 1 (TBK1) is a non-canonical IκB kinase that contributes to KRAS-driven lung cancer. Here we report that TBK1 plays essential roles in mammalian cell division. Specifically, levels of active phospho-TBK1 increase during mitosis and localize to centrosomes, mitotic spindles and midbody, and selective inhibition or silencing of TBK1 triggers defects in spindle assembly and prevents mitotic progression. TBK1 binds to the centrosomal protein CEP170 and to the mitotic apparatus protein NuMA, and both CEP170 and NuMA are TBK1 substrates. Further, TBK1 is necessary for CEP170 centrosomal localization and binding to the microtubule depolymerase Kif2b, and for NuMA binding to dynein. Finally, selective disruption of the TBK1–CEP170 complex augments microtubule stability and triggers defects in mitosis, suggesting that TBK1 functions as a mitotic kinase necessary for microtubule dynamics and mitosis.

## Abstract (web summary)

TANK binding kinase 1 (TBK1) is a non-canonical IκB kinase that regulates immunity via NF-κB. Here Pillai *et al*. show that TBK1 localizes to centrosomes during mitosis, and regulates microtubule dynamics and spindle formation by phosphorylating the centrosomal protein CEP170 and the mitotic apparatus protein NuMa.

## Graphical abstract

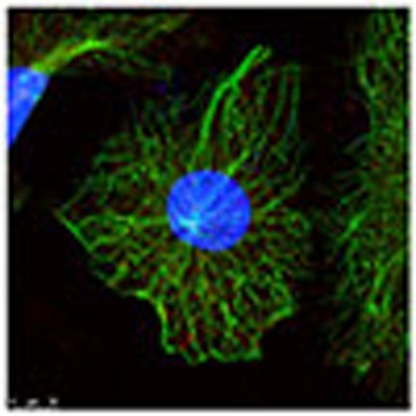

TBK1 is an IKK (IκB Kinase)-related kinase that is activated by phosphorylation of Serine-172 by TLR and RIG1 signalling, and this circuit triggers phosphorylation of IRF3 and IRF7, activation of NFκB and the expression of proinflammatory genes and interferons^1^,^2^,^3^,^4^,^5^,^6^. In addition to the crucial role TBK1 plays in regulating innate immunity, recent studies suggest that TBK1 participates in pathways leading to survival and cellular transformation^7^. RalB-mediated activation of TBK1 promotes TBK1 assembly with the exocyst complex through its interaction with Sec5 leading to inflammatory responses and prosurvival signalling by directly phosphorylating multiple sites on Akt^8^. TBK1 is essential for the survival of non-small cell lung cancers driven by oncogenic KRAS^9^,^10^,^11^; this synthetic lethal interaction of TBK1 with mutant K-Ras was governed by its ability to activate NFκB anti-apoptotic signalling through c-Rel and BCL-XL. TBK1 also contributes to prostate cancer dormancy and drug resistance by inhibiting mTOR^12^, and to tamoxifen resistance of breast cancer cells by enhancing transcriptional activity of ERα^7^.

TBK1 has been reported to phosphorylate the mitotic kinase PLK1 (ref. 13), but roles for TBK1 in mitosis have not been investigated. Here we demonstrate direct roles for TBK1 in regulating mitosis, where it binds to and phosphorylates CEP170, a forkhead domain and centrosome- and spindle microtubule-associated protein^14^, as well as NuMA, which associates with the pericentrosomal domains of the spindle apparatus and is necessary for cytokinesis^15^. Here we demonstrate that TBK1 regulates microtubule dynamics and also mitotic progression by modulating CEP170 and NuMA functions.

## Results

### pS172 TBK1 localizes to centrosomes and mitotic spindles

**Figure 1:**
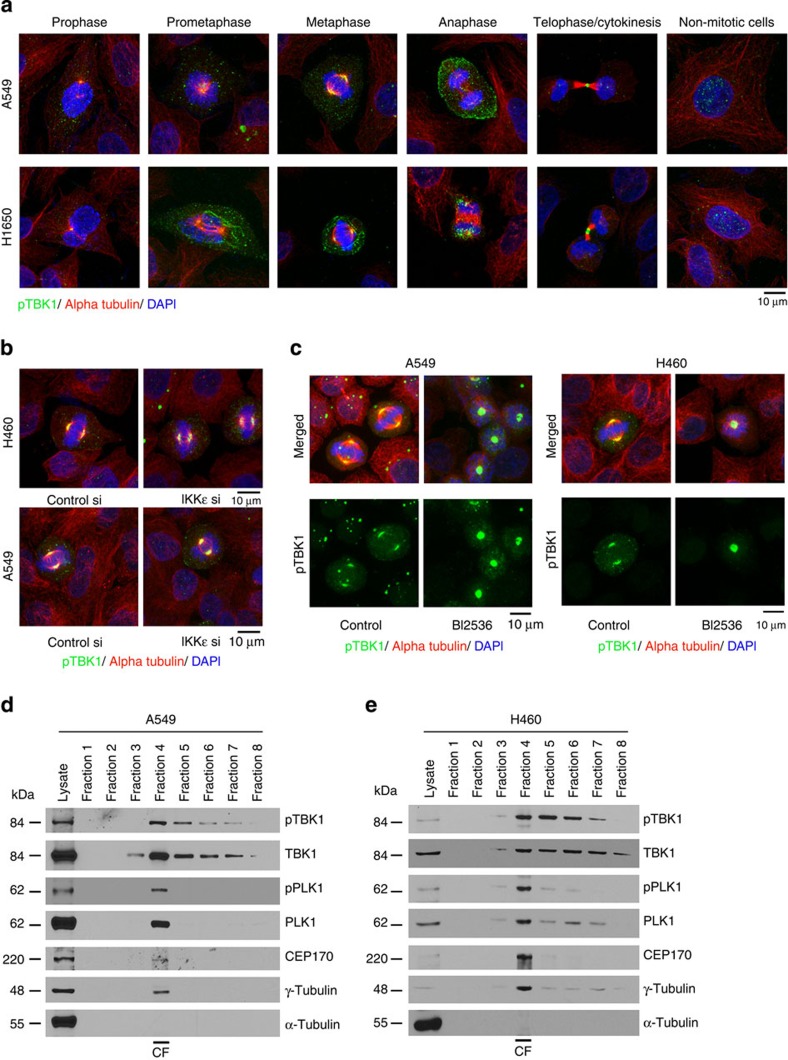
Phospho-S172 TBK1 localizes to centrosomes and mitotic spindles. (**a**) Confocal microscopy images of A549, H1650 and H460 cells progressing through different mitotic stages. Cells were stained for phospho-TBK1 (green), alpha tubulin (red) and DNA (DAPI, Blue). Scale bar, 10 μm. (**b**) Confocal microscopy images from H460 and A549 cells stained for phospho-TBK1 (green), alpha tubulin (red) and DNA (DAPI, Blue) after depleting IKKɛ using siRNAs (**c**) PLK1 using the inhibitor BI2536 in H460 and A549 cells. A non-targeting control siRNA was used as control. Depletion of IKKɛ or inhibition of PLK1 does not affect the localization of pTBK1 and alpha tubulin. (**d**,**e**) Western blots for pTBK1, TBK1, pPLK1, PLK1, CEP170, γ-tubulin and alpha tubulin from different fractions collected during centrosome isolation from H460 and A549 cells. pTBK1 and TBK1 are enriched in the centrosomal fraction (Fraction-4). Purity of the centrosomal fraction is indicated by high levels of γ-Tubulin in Fraction-4. CF indicates the centrosomal fraction. Lysate lane represents whole cell lysate prepared from asynchronously growing H460 or A549 cells. Experiments were repeated three times and representative images are presented.

Immunofluorescence experiments using a phospho-TBK1 (pS-172) specific antibody on A549, H1650, Calu-6 and PC9 non-small cell lung cancer (NSCLC) cell lines as well as the immortalized human tracheobronchial epithelial cell line AALE established that phospho-TBK1 localized to centrosomal regions during prophase and prometaphase, where it co-localized with alpha tubulin (Fig. 1a, Supplementary Fig. 1a–d). Similar findings were manifest in U937 myeloid leukaemia cells and Daudi Burkitt lymphoma cells (Supplementary Fig. 1e), and a second phospho-TBK1 antibody showed similar localization of pTBK1 (Supplementary Fig. 2). Further, phospho-TBK1 associated with spindle microtubules during metaphase and with the midbody during telophase and cytokinesis (Fig. 1a). Finally, depletion of TBK1-related IKKɛ kinase using siRNAs (Fig. 1b) or inhibition of mitotic kinase PLK1 using the inhibitor BI2536 (Fig. 1c) did not alter the centrosomal localization of phospho-TBK1.

To further confirm the centrosomal localization of phospho-TBK1, centrosomes were isolated from A549 and H460 NSCLC cells by discontinuous sucrose gradient fractionation^16^,^17^ and subjected to western blot analysis. Phospho-TBK1 and total TBK1 were principally found in centrosomal fraction (Fraction 4, Fig. 1d,e), which also contained γ-tubulin, phospho-PLK1, PLK1 and CEP170. Phospho-TBK1 and total TBK1 were also present in additional fractions; correlating with the observation that phospho-TBK1 also is associated with spindle apparatus during mitosis. Interestingly, pTBK1 localization to centrosomes did not depend on microtubule integrity, as pTBK1 localized to centrosomes when microtubules were hyperstabilized or depolymerized (Supplementary Fig. 3).

### TBK1 is necessary for progression through mitosis

**Figure 2:**
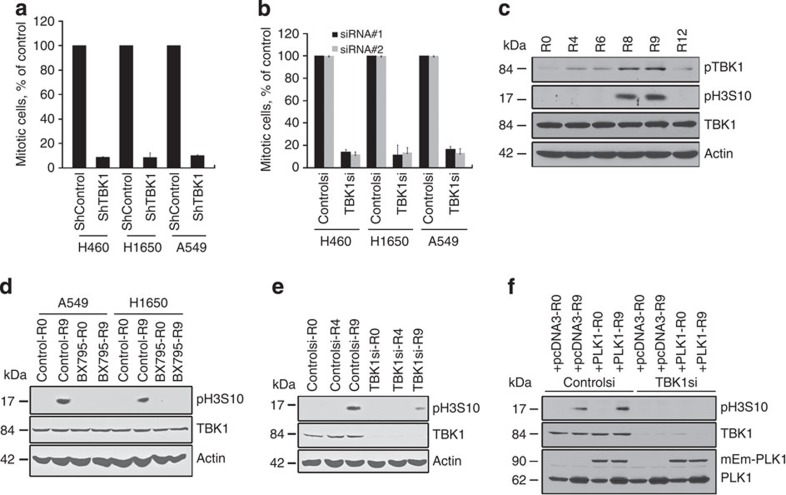
Inhibition or silencing of TBK1 induces mitotic defects and inhibits mitosis. (**a**) Knockdown of TBK1 by transfecting two different siRNAs in A549 and H1650 cells resulted reduces the number of mitotic cells. Control siRNA refers to transfection with a non-targeting siRNA, (**b**) Knockdown of TBK1 using lentiviral shRNA in H1650, H460 and A549 cells reduces the number of mitotic cells, For **a**,**b**, error bars represent standard deviation (s.d.) *P<0.05; *t*-test was applied. Experiments were repeated three times. (**c**) Western blot analysis of cells synchronized at G1/S by double-thymidine treatment and released for 2, 4, 6, 8 or 12 h; phospho-TBK1, Phospho- S172 TBK1; TBK1, total TBK1; pH3S10, histone H3 phosphorylated at serine 10 (mitotic marker). (**d**) Western blot showing the levels of Histone H3 phosphorylated at serine 10 after TBK1 inhibition by BX795 treatment (0.5 μM) in H1650 and A549 cells. Cells were synchronized to G1/S by double-thymidine block and subsequently allowed to proceed to mitosis. R0, synchronized by double-thymidine block; R4, released for 4 h, R9 released for 9 h. (**e**) Western blot showing the levels of Histone H3 phosphorylated at serine 10 (H3pS10) after TBK1 depletion by siRNA transfection in A549 cells. (**f**) Western blots showing the levels of phospho histone H3 S10 (pH3S10), to test if overexpression of PLK1 can overrideTBK1 depletion. For this, A549 cells were transfected with control or TBK1 siRNA along with a control plasmid (pcDNA3) or mEmerald-PLK1 to overexpress PLK1. Following transfection, cells were subjected to double-thymidine block and then released to enter mitosis. Levels of pH3S10, TBK1 and PLK1 were assessed by western blotting. Control siRNA-treated cells showed elevated levels of pH3S10 when released from G1/S block, indicating entry into mitosis; in contrast, TBK1-depleted cells did not show high levels of pH3S10 even when PLK1 was overexpressed, suggesting PLK1 cannot rescue mitotic arrest triggered by TBK1 depletion.

Given the centrosomal localization of phospho-TBK1, we assessed if TBK1 contributes to mitosis. Depletion of TBK1 by two different TBK1-selective siRNAs or by a lentiviral small-hairpin RNA (shRNA; Supplementary Fig. 4) significantly reduced the number of mitotic cells (Fig. 2a,b).

Centrosomal structures start to get organized towards the end of the S-phase^18^. To assess if this was associated with localization of active phospho-TBK1 to centrosomes, A549 NSCLC cells were arrested at the G1/S transition by double-thymidine block. Release from arrest showed that TBK1 is activated at late S-phase, 4 h after release from the double-thymidine block (Fig. 2c). Finally, maximal levels of phospho-TBK1 phosphorylation coincided with increased levels of phosphorylation of histone H3 at serine 10 (pH3S10), an indicator for progression into mitosis (Fig. 2c).

To test whether TBK1 inhibition prevents cell cycle progression, A549 and H1650 NSCLC cells were arrested in the G1/S phase transition by double-thymidine block and released for 9 h in the presence or absence of BX795. Untreated cells, but not BX795-treated cells, entered mitosis as seen by elevated levels of pH3S10 in western blots (Fig. 2d). Similarly selective knockdown of TBK1 using siRNAs prevented entry of cells into mitosis following double-thymidine block and release, as seen by low levels of pH3S10 (Fig. 2e).

A recent study suggested that PLK1, a well-established mitotic kinase, might be a TBK1 substrate^13^. Hence, we tested whether TBK1 regulates mitosis via PLK1, and whether PLK1 overexpression could override the effect of TBK1 knockdown. A549 NSCLC cells engineered to overexpress PLK1 were treated with siRNA targeting TBK1, and were then subjected to double-thymidine block and released. Analyses of histone H3 phosphorylation established that TBK1 knockdown compromised entry into mitosis regardless of PLK1 overexpression (Fig. 2f). Thus, PLK1 cannot compensate for the mitotic functions directed by TBK1 and TBK1 must target additional proteins to modulate mitosis.

### Inhibition of TBK1 increases 4*n*+ cells

**Figure 3:**
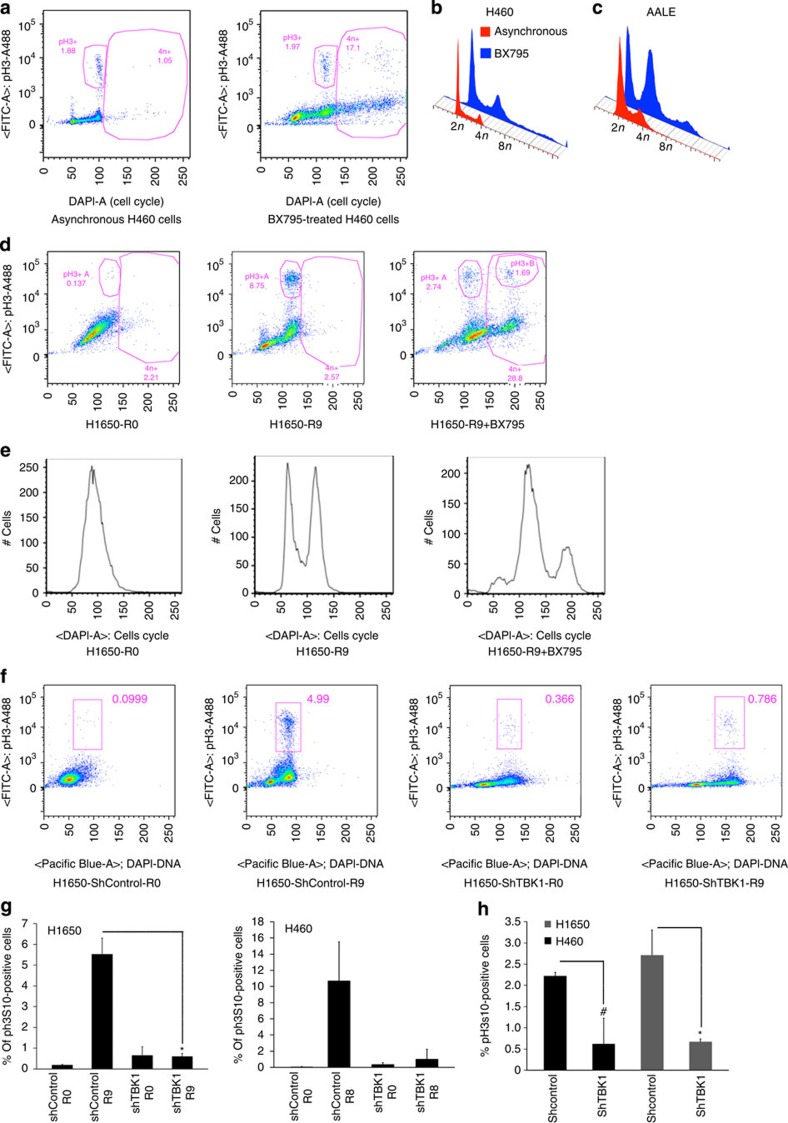
Inhibition of TBK1 affects mitosis and enhances 4*n*+ population. (**a**) Cell cycle analysis of asynchronously growing H460 cells in the presence or absence of BX795 by flow cytometry. (**b**,**c**) Histograms summarizing cell cycle analysis of control and BX795-treated asynchronously growing H460 (**b**) and AALE cells (**c**). (**d**,**e**) Cell cycle analysis combined with pH3S10 staining of synchronized H1650 cells released for 9 h in the presence of BX795 by flow cytometry. (**f**) Cell cycle analysis of H1650 shcontrol cells (transduced with lentivirus containing scrambled sequence) or H1650 shTBK1 cells (transduced with lentivirus containing shRNA targeting TBK1) synchronized by double-thymidine block and released for 9 h by flow cytometry. In panels a, d and e, the *y* axis shows the quantification of cells stained for H3pS10 and the *x* axis shows DNA content (DAPI staining). (**g**) Graphical representation of the flow cytometry data showing reductions in the number of phospho histone H3 positive/mitotic cells when TBK1 is depleted in H1650 and H460 cells. (*P<0.05, *t*-test) (**h**) Graphs indicating the inhibition of cells undergoing mitosis when TBK1 was depleted in asynchronously growing H1650 and H460 cells. (*P<0.05; ^#^*P*<0.067; *t*-test). Error bars represent s.d. Two replicates are included in these experiments.

To confirm a role for TBK1 in cell division, control cells and BX795-treated cells were analysed for changes in H3pS10 levels and DNA content by bivariate flow cytometry. Treatment of asynchronously growing cells with the TBK1 inhibitor BX795 triggered marked (over 15-fold) increases in the proportion of 4*n*+ H460 cells (1.05 to 17.1%, Fig. 3a,b) and AALE cells (4.98 to 19%; Fig. 3c). Similarly, BX795 treatment of double-thymidine block/released H1650 NSCLC cells led to over threefold reduction in the proportion of cells expressing phospho-H3S10, and to >11-fold increase in the percentage of cells with 4*n*+ DNA content (from 2.57 to 28.8%, Fig. 3d,e). Reduction in the proportion of cells entering mitosis was also observed when H1650 cells were transfected with a lentiviral vector expressing shRNA to TBK1 (Fig. 3f) and released from double-thymidine block. Confocal immunofluorescence analyses of phospho-H3S10 showed that similar effects were manifested in synchronized (Fig. 3g) or asynchronous H1650 and H460 NSCLC cells following TBK1 knockdown (Fig. 3h). These results suggest that TBK1 plays essential roles in mitotic progression.

### TBK1 is necessary for completion of mitosis

**Figure 4:**
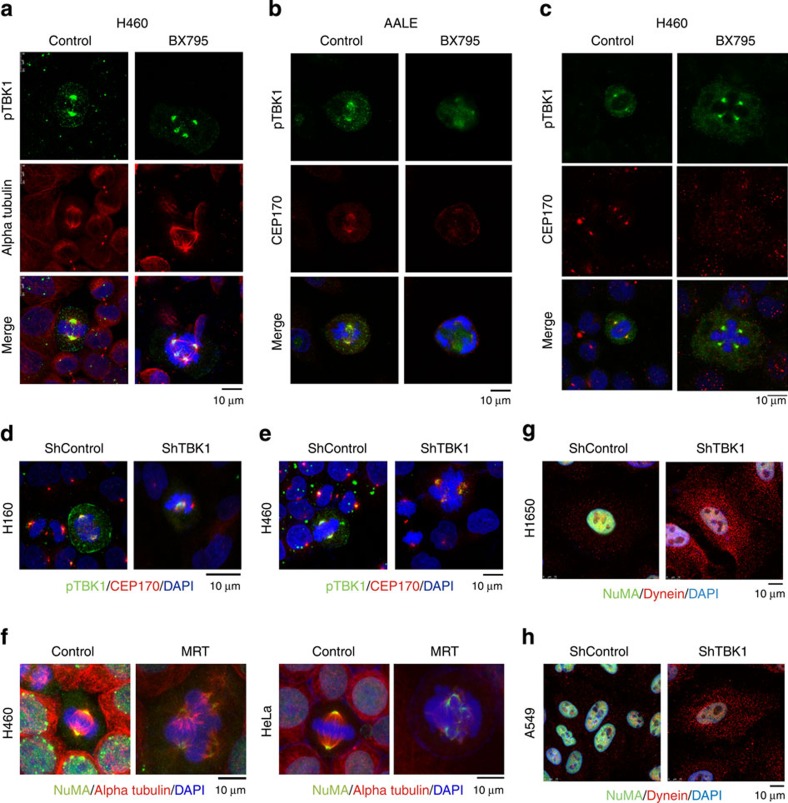
Phospho-TBK1 co-localizes with CEP170 and TBK1 inhibition provokes supernumerary centrosomes. (**a**) Confocal microscopy images of control and BX795-treated H460 cells at metaphase. Cells were stained for phospho-TBK1 (green), alpha tubulin (red) and DNA (DAPI, blue). Scale bar, 10 μm. (**b**) Confocal microscopy images of control and BX795-treated AALE cells at metaphase. Cells were stained for phospho-TBK1 (green), CEP170 (red) and DNA (DAPI, blue). Scale bar, 10 μm. (**c**) H460 cells stained for phospho-TBK1 (green), CEP170 (red) and DNA (DAPI, blue) in control and TBK1 inhibitor-treated cells. CEP170 fails to co-localize to centrosomes in BX795-treated cells. (**d**,**e**) TBK1 depletion by shRNA resulted in multiple centrosomes in H1650 (**d**) and H460 (**e**) cells. Cells were stained for phospho-TBK1 (green), CEP170 (red) and DNA (DAPI, blue). (**f**) Inhibition of TBK1 by 2 μM MRT affected spindle pole localization of NuMA. H460 and HeLa cells were stained for NuMA (green), Alpha Tubulin (red), DAPI (Blue). Scale bar, 10 μm. (**g**,**h**) Immunofluorescence staining for Dynein intermediate chain (red) and NuMA (green) in shcontrol and shTBK1-transfected H1650 (**g**) and A549 (**h**) cells. Scale bar, 10 μm.

We next tested if TBK1 inhibition by BX795 provoked mitotic defects or spindle abnormalities. Confocal microscopy analyses of BX795-treated H460 NSCLC cells revealed a large number of cells with more than two centrosomes, indicative of either centrosome amplification or alteration in cell division (Fig. 4a). Supernumerary centrosomes can arise due to a number of mechanisms, including centrosome overduplication, defects in cell division, cell fusion or *de novo* genesis^19^. Live cell imaging analyses of H460 cells treated with BX795 or MRT67307 revealed that TBK1 inhibition resulted in inhibition of mitotic progression beyond metaphase leading to the formation of multinucleate cells (Supplementary Movies 1,2 and 3). We observed that certain cells appeared to revert back from a mitotic state, while certain others underwent cell death. Live cell imaging on HeLa cells transfected with GFP-tagged alpha tubulin and mCherry-tagged histone H2B confirmed that cells treated with either BX795 or MRT67307 were unable to progress beyond metaphase, where treated cells attempted and failed to align the chromosomes in metaphase plate (Supplementary Movies 4,5 and 6). Thus, the formation of supernumerary centrosomes following TBK1 inhibition is at least partly due to a failure in completion of cell division.

We further examined if inhibition of TBK1 contributes to centrosome amplification. For this, U2OS cells were treated either with 2 mM hydroxyurea (HU) or TBK1 inhibitor BX795 or both for 72 h and subsequently immunostained with γ-tubulin antibody to visualize and quantify number of centrosomes in each cell. When cells are treated with DNA synthesis inhibitors such as HU, it results in uncoupling of DNA replication and centrosome duplication, resulting in three or more centrosomes per cell. 40% HU-treated cells exhibited three or more centrosomes, while TBK1 inhibitor alone could induce centrosome duplication in 22% of cells. Interestingly, both HU and BX795 induced centrosome duplication in almost 58.7% of cells (Supplementary Fig. 5); hence it is tempting to speculate that different mechanisms might regulate HU-induced and TBK1 inhibitor induced centrosome amplification. It is also possible that the increase in centrosome number after TBK1 Inhibition might be a consequence of failure to complete cell division.

### TBK1 associates with and phosphorylates CEP170 and NuMA

Confocal microscopy analyses revealed that active phospho-TBK1 localized to centrosomes and spindle fibres during mitosis (Fig. 4b). This suggested that active TBK1 might directly bind to and phosphorylate phosphoproteins that are components of centrosomes and/or the spindle apparatus. Indeed, double immunofluorescence analyses showed that CEP170, a centrosomal protein^14^,^20^, co-localized with phospho-TBK1 on centrosomes in AALE and H460 cells. Similar findings were also evident in mitotic U937 myeloid leukaemia and Daudi Burkitt's lymphoma cells (Supplementary Fig. 6).

Notably, inhibition of TBK1 activity by treatment of NSCLC cells with BX795 (Fig. 4b,c), or shRNA-directed knockdown of TBK1 (Fig. 4d,e, Supplementary Fig. 7), affected mitosis and the association of CEP170 with centrosomes. Further, TBK1 inhibition affected spindle pole localization of NuMA, a phosphoprotein that associates with the minus-end-directed molecular motor protein dynein during mitosis^20^,^21^,^22^,^23^ (Fig. 4f, Supplementary Fig. 8). Finally, TBK1 knockdown or inhibition also affected the levels of cytoplasmic dynein (Fig. 4g,h, Supplementary Fig. 9), suggesting that TBK1 kinase activity is necessary for proper centrosomal localization of CEP170 and NuMA during mitosis.

**Figure 5:**
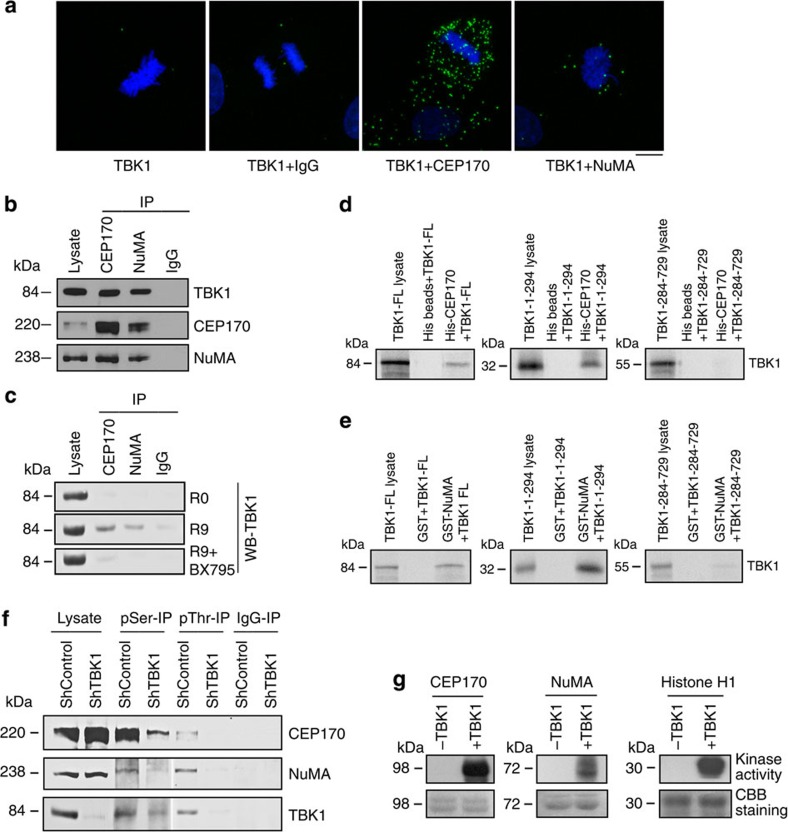
TBK1 directly binds to and phosphorylates CEP170 and NuMA. (**a**) Detection of co-localization of TBK1 and CEP170 as well as TBK1 and NuMA using proximity ligation assay (PLA). Green fluorescent foci represent co-localization of TBK1 and CEP170 or TBK1 and NuMA. TBK1 antibody alone or TBK1 antibody along with IgG were used as negative controls. (**b**) Immunoprecipitation–western blot analysis showing the interaction of endogenous TBK1 with endogenous CEP170 and NuMA in lysates of asynchronously growing H1650 cells. (**c**) Immunoprecipitation–western blot analysis of H460 cell lysates showing the binding of TBK1 with NuMA and CEP170 when released from G1/S block. This interaction was inhibited when cells were released from double-thymidine block in the presence of BX795. (**d**,**e**) *In vitro* binding of full-length TBK1, the kinase domain of TBK1 (1–294) or the TBK1 C terminus (TBK1-284-729) with CEP170 (**d**) or NuMA (**e**). The data presented are representative of three independent experiments. Lysate/ input lane represents 20% of the input ^35^S-methionine-labelled TBK1 used in the pull-down assay. (**f**) Immunoprecipitation–western blot analysis showing inhibition in serine and threonine phosphorylation of CEP170 and NuMA when TBK1 was depleted following transduction of cells with lentivirus expressing shRNA targeting TBK1. (**g**) *In vitro* kinase assays showing the phosphorylation of NuMA and CEP170 by TBK1. Coomassie brilliant blue (CBB) staining shows the amount of protein in each reaction.

Proximity ligation assay (PLA) is a relatively novel technique to accurately detect and quantify protein–protein interactions in cells and tissue sections^21^. Primary antibodies from different species together with secondary antibodies specific for immunoglobulins from corresponding species modified by attachment of DNA strands, are used as proximity probes to generate circular DNA strands. Signal amplification by replicating the DNA circles via rolling circle amplification reveals proteins in close proximity (<40-nm apart) as distinct foci. Proximity ligation assays using the Duolink system^22^ showed co-localization of TBK1 and CEP170 and of TBK1 and NuMA, compared with control samples that were stained with only one antibody or a normal IgG (Fig. 5a). To assess if these proteins physically interact, co-immunoprecipitation–western blot experiments were conducted on the endogenous proteins. Indeed, TBK1 could interact with CEP170 and NuMA in H1650 NSCLC cells (Fig. 5b) and this interaction was augmented during mitosis and abolished by treatment with BX795 (Fig. 5c). Finally, full length ^35^S-labelled TBK1 could specifically bind to recombinant GST-NuMA and His-CEP170 proteins conjugated on sepharose beads, but not to beads primed with GST alone, or to unprimed His-sepharose beads (Fig. 5d,e). Finally, *in vitro* binding assays also showed that ^35^S-labelled TBK1 kinase domain (TBK1-1-294) but not the C terminus of TBK1 (residues 284–729) directed binding to CEP170 and NuMA (Fig. 5d,e).

Collectively, these findings suggested that CEP170 and NuMA might be TBK1 substrates. In support of this notion, immunoprecipitation using anti-phosphoSerine or anti-phosphoThreonine antibody and subsequent western blot analyses established that knockdown or inhibition of TBK1 led to marked reductions in the levels of phospho-CEP170 and phospho-NuMA (Fig. 5f). Finally, *in vitro* kinase assays using purified His-tagged CEP170 or GST-NuMA as substrates showed that purified TBK1 could efficiently phosphorylate CEP170 and NuMA (Fig. 5g), and mass spectrometry analysis revealed that TBK1 phosphorylated 12 serine residues and a threonine residue of CEP170, as well as five serine residues of NuMA (Supplementary Tables 1 and 2).

### TBK1 controls microtubule dynamics during mitosis

**Figure 6:**
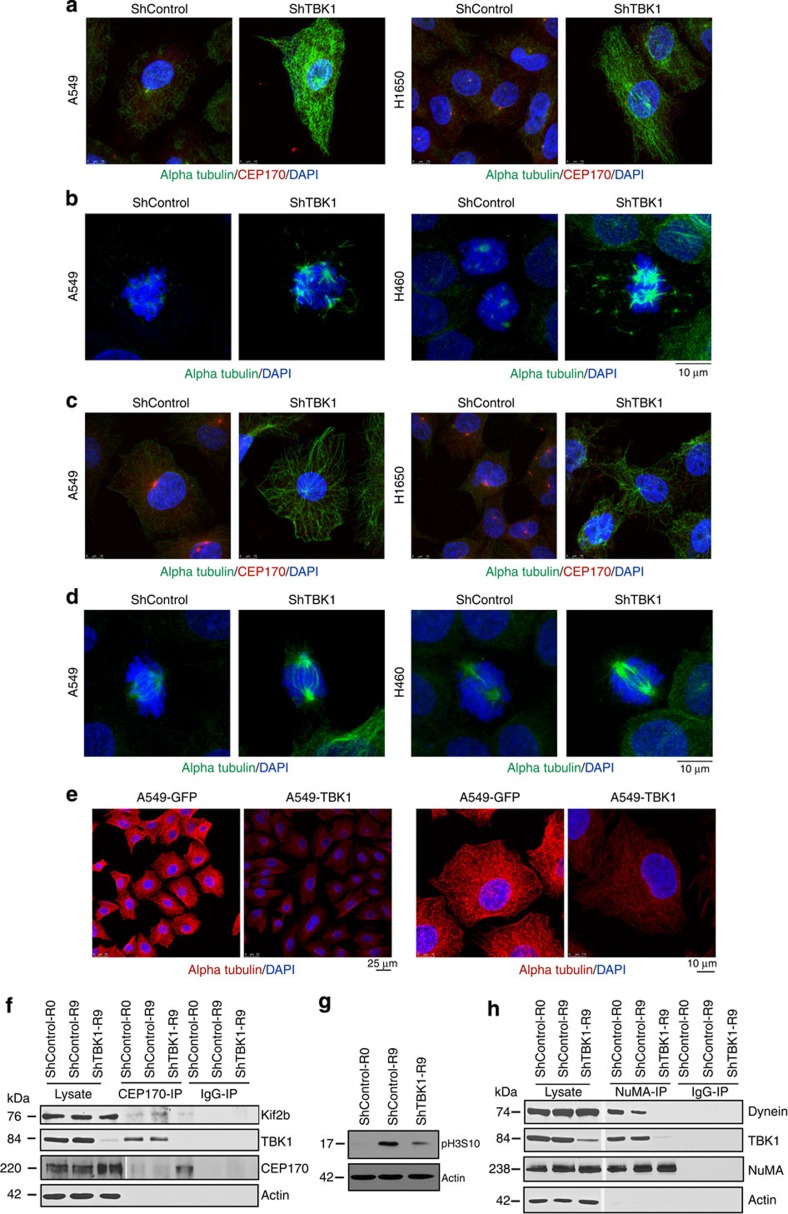
TBK1 depletion alters microtubule dynamics. (**a**) Shcontrol A549 or H1650 cells treated with 0.1 μM nocodazole for 15 min showed complete disruption of microtubules, while TBK1-depleted shTBK1 A549 or H1650 cells retained stable microtubules as seen by staining for alpha tubulin (green) and CEP170 (red). (**b**) Mitotic spindles in TBK1-depleted A549 and H460 cells were more stable after nocodazole treatment as compared to control cells. Alpha tubulin (green) shows mitotic spindles. (**c**) shTBK1 A549 or H1650 cells when subjected to cold temperature (4 °C) and subsequent incubation at warm temperature (37 °C) had stable microtubules versus analogously treated shcontrol A549 or H1650 cells. Alpha Tubulin (green), CEP170 (red) and DNA (DAPI, Blue). (**d**) Similar results were observed in mitotic cells where mitotic spindles of TBK1-depleted A549 or H460 cells were more stable compared to control shRNA-treated cells. Alpha tubulin (green). (**e**) Stability of microtubules were drastically reduced in A549 cells overexpressing TBK1 when treated with 5 μM nocodazole and allowed to regrow for 7 min; Scale bar, 10 μm for **e**. (**f**) Low magnification images of cells in **c**. Scale bar, 25 μm. (**g**–**i**) TBK1 is required for the binding of CEP170 with Kif2b (**g**) as well as for the association of NuMA with dynein (**i**), as seen by immunoprecipitation–western blot analysis. (**h**) Western blot for pH3S10 to confirm mitotic fractions.

Since TBK1 depletion or inhibition provoked the formation of multiple mitotic spindle structures in the cell, we assessed if TBK1 regulates microtubule stability and dynamics. To test this, shRNA targeting TBK1 or a non-targeting control shRNA were transduced in A549 and H1650 cells, which were then treated with 0.1 μM nocodazole for 15 min (refs 23, 24). Immunofluorescence analyses of interphase cells using alpha tubulin antibody showed that microtubules were completely depolymerized by nocodazole in control shRNA-treated cells (Fig. 6a), but not in TBK1-depleted cells. Similarly, mitotic spindles were more stable in TBK1-depleted mitotic cells as compared with control shRNA-treated mitotic cells (Fig. 6b). To assess if TBK1 regulates regrowth of microtubules, control and TBK1-depleted cells were shifted to cold temperature to depolymerize the microtubules, and then shifted back to 37 °C to allow regrowth of microtubules^23^,^25^. Again, confocal immunofluorescence analyses using an alpha tubulin antibody established that TBK1 depletion results in more stable and polymerized microtubules in both H1650 and A549 NSCLC interphase cells (Fig. 6c). Similar trend was observed in mitotic cells subjected to microtubule depolymerization and subsequent regrowth (Fig. 6d). Finally, to test if overexpression of TBK1 compromised microtubule stability, A549 NSCLC cells engineered to overexpress TBK1 (A549-TBK1) and control (A549-GFP) cells were treated with nocodazole (5 μM) for 1 h to depolymerize microtubules; nocodazole was then removed to allow microtubule polymerization^26^. Control cells showed polymerized microtubule network, while polymerization was significantly reduced in TBK1-overexpressing cells (Fig. 6e; low- and high-resolution images are shown).

The dynamic nature of microtubules ensures correct chromosome segregation during mitosis and this is modulated by microtubule-associated proteins^24^, including kinesins and dyneins^27^,^28^,^29^,^30^. The kinesin 13 family member Kif2b stably associates with CEP170 and this interaction enhances its targeting to the mitotic spindle^31^. Hence, we examined whether TBK1-mediated phosphorylation of CEP170 affects its interaction with Kif2b. Control or TBK1-depleted cells were synchronized by double-thymidine block and the presence of Kif2b in CEP170 immunoprecipitates was examined by western blotting. Notably, the interaction between CEP170 and Kif2b was significantly reduced in TBK1-depleted cells (Fig. 6f); pH3S10 was reduced in shTBK1-transfected cells, as expected (Fig. 6g). Finally, immunoprecipitation–western blot analysis also revealed that the interaction of dynein with NuMA was impaired following knockdown of TBK1 (Fig. 6h). Collectively, these findings indicate that TBK1 is necessary for proper microtubule dynamics during mitosis.

### Binding of TBK1 to CEP170 is needed for mitotic progression

**Figure 7:**
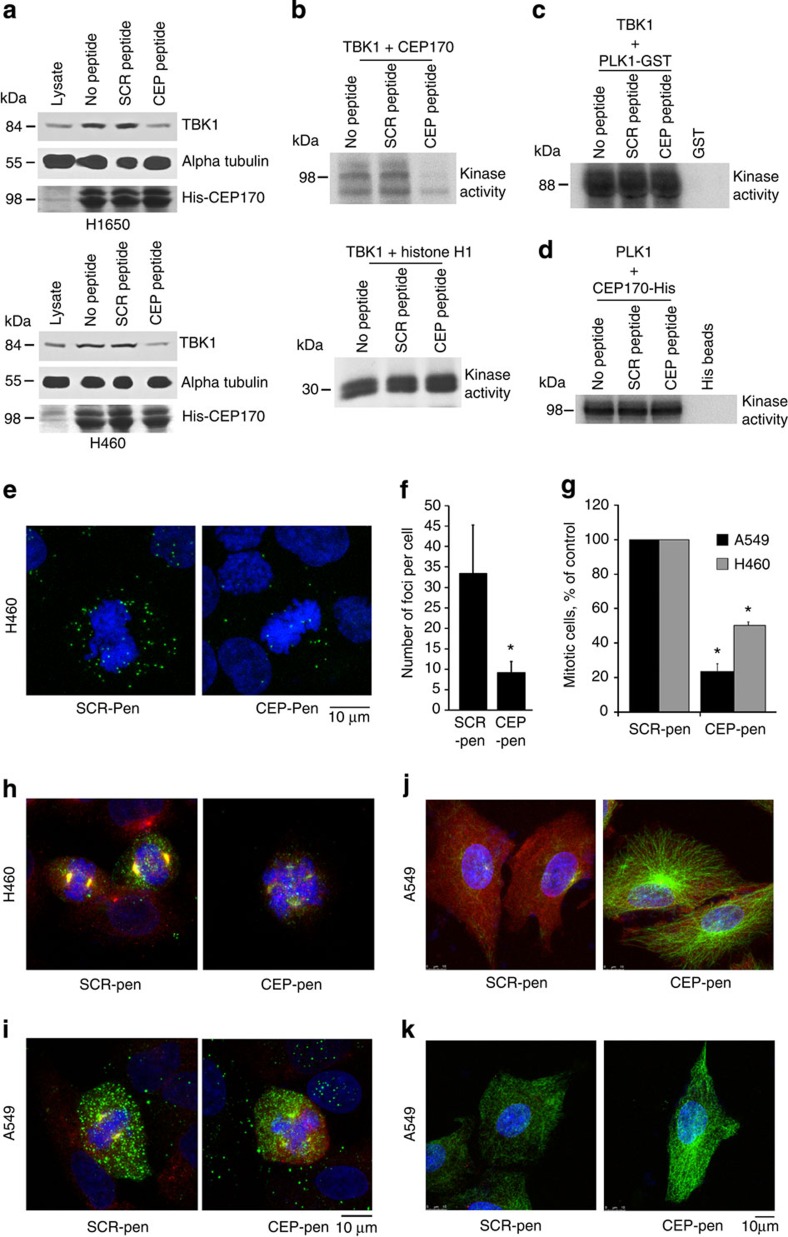
CEP peptide disrupts binding of CEP170 and TBK1 and provokes mitotic defects. (**a**) H460 or H1650 cell lysates were incubated with 5 μM CEP or scrambled peptide along with His-tagged CEP170 beads. Western blotting shows that binding of TBK1 to CEP170 was specifically disrupted by the CEP peptide, but binding of His-CEP170 to alpha tubulin was not. (**b**) Scrambled or CEP peptides (1 μM) were preincubated with TBK1 for 15 min and used in a kinase reaction with His6-CEP170 as a substrate. *In vitro* kinase assay showed that the CEP170 peptide effectively inhibits His-CEP170 phosphorylation by TBK1 but not the phosphorylation of Histone H1. (**c**) *In vitro* kinase assays showing that CEP-peptide specifically inhibits TBK1-mediated phosphorylation of CEP170 but not PLK1, where a kinase dead PLK1 was used as substrate. (**d**) PLK1-mediated phosphorylation of CEP170 is not affected by CEP170-peptide in kinase assays; His6-tagged CEP170 was used as a substrate. (**e**) CEP170 peptide conjugated to penetratin (CEP-Pen) inhibited the CEP170–TBK1 interaction in H460 cells but a scrambled peptide–penetratin conjugate did not, as seen by a proximity ligation assay. Green signal indicate points of co-localization between TBK1 and CEP170. Scale bar, 10 μm. (**f**) Quantification of signal from cells treated with peptides (*=*P*<0.05, t test; Error bars represent s.d. Two replicates were included). (**g**) H1650 or H460 cells treated with CEP peptide conjugated to penetratin (CEP-Pen) showed reduced the number of mitotic cells versus those treated with scrambled peptide–penetratin conjugate (SCR-Pen) (*P<0.05, *t* test; Error bars represent s.d. Two replicates were included). (**h**,**i**) H460 and A549 cells treated with CEP-penetratin conjugate showed mitotic defects. Phospho-TBK1 (green), CEP170 (red) and DNA (DAPI, Blue). Scale bar, 10 μm. (**j**) A549 cells treated with CEP-Pen subjected to cold temperature (4 °C) and shifted to 37 °C resulted in more stable microtubules compared to SCR-Pen-treated cells. Alpha tubulin (green), CEP170 (red) and DNA (DAPI, Blue). (**k**) Microtubules were more stable in A549 cells treated with CEP-Pen when exposed to 0.1 μM nocodazole for 15 min versus SCR-Pen treated cells. Alpha tubulin (green), CEP170 (red) and DNA (DAPI, Blue). Scale bar, 10 μm.

To test whether the TBK1–CEP170 interaction was necessary for mitosis, we synthesized a 13 amino-acid peptide corresponding to a region of CEP170 that was phosphorylated by TBK1 (CEP peptide ENAESEADFSIHF), which we predicted would competitively inhibit CEP170 binding to TBK1; a scrambled peptide derived from this sequence was also generated (Scr peptide AFIFAHEESEDNSC). *In vitro* binding assays with His-tagged CEP170 beads and lysates of H1650 or H460 NSCLC cell lysates confirmed that 5 μM CEP peptide selectively inhibited the interaction of CEP170 with endogenous TBK1 (Fig. 7a, upper and lower panels). Further, binding of His-CEP170 to alpha tubulin was not affected by CEP peptide, showing its specificity. Finally, CEP peptide (1 μM; but not the Scr peptide) effectively inhibited phosphorylation of His-CEP170 by TBK1, but not TBK1-directed phosphorylation of Histone H1 (Fig. 7b).

Since TBK1 can phosphorylate PLK1 and since CEP170 is a PLK1 target, we tested whether the CEP peptide would inhibit TBK1-mediated phosphorylation of PLK1, or CEP170 phosphorylation by PLK1. *In vitro* kinase assays conducted using TBK1 with PLK1-GST as the substrate in the presence of the scrambled or CEP peptide showed that phosphorylation of PLK1 by TBK1 was not affected by the presence of the CEP peptide (Fig. 7c). Similarly, the CEP peptide did not inhibit the phosphorylation of CEP170-His by PLK1 kinase (Fig. 7d). Thus, the CEP peptide can specifically inhibit the association of TBK1 with CEP170 and its phosphorylation by TBK1.

To test whether CEP peptide could inhibit binding of TBK1 to CEP170 in cells, proximity ligation assays were performed. The above peptides were conjugated to the carrier molecule penetratin^32^,^33^, to facilitate their delivery into the cells, and the effect of the interaction of TBK1 with CEP170 was assessed. These assays showed a significant reduction in the binding of TBK1 to CEP170 in H460 NSCLC cells treated with the CEP170 peptide–penetratin conjugate (Fig. 7e), which was also evident by the reduced number of foci in CEP-peptide-treated cells (average of 9 foci per cell) versus Scr-peptide-treated cells (33 foci per cell) (Fig. 7f). Further, treatment with the CEP170 peptide conjugate, but not the scrambled peptide conjugate, significantly reduced the number of mitotic cells (Fig. 7g) as well as the interaction of TBK1 with CEP170 on the centrosomes (Fig. 7h,i). Supporting the above results, studies assessing microtubule regrowth on cells treated with the CEP170 peptide conjugate showed marked stabilization of microtubules, comparable to that observed following inhibition or depletion of TBK1 (Fig. 7j). Finally, treatment of NSCLC cells with the CEP170 peptide conjugate led to microtubule stabilization following nocodazole treatment (Fig. 7k). We conclude the TBK1–CEP170 interaction is necessary for both microtubule dynamics and mitosis.

### Phosphorylation site mutants of CEP170 and NuMA induce mitotic defects

**Figure 8:**
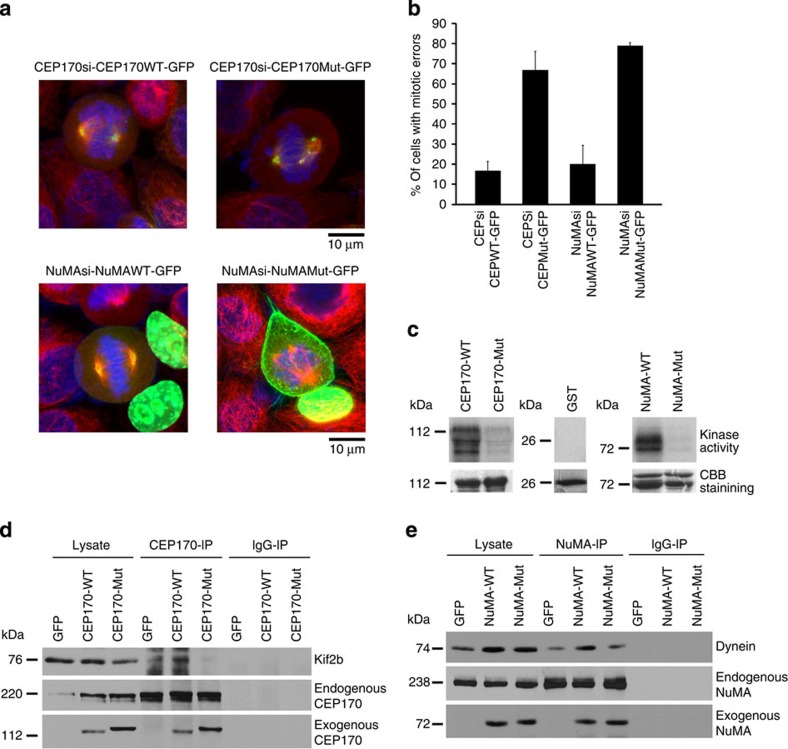
Phosphorylation site mutants of CEP170 and NuMA induce mitotic defects. (**a**) HeLa cells were transfected with CEP170 or NuMA siRNA to deplete the endogenous levels of these proteins and subsequently transfected with GFP-tagged wild-type or mutant constructs of CEP170 or NuMA. Cells were fixed and subsequently stained for Alpha tubulin (red). Mutant CEP170 or mutant NuMA transfected cells showed mitotic defects compared to wild-type CEP170 or NuMA transfected cells. (**b**) Quantification of cells with mitotic defects. Graphs represent average of two experiments. Error bars represent s.d. (**c**) Kinase assay with wild-type or mutant GST tagged CEP170 or NuMA as substrates. Images shown are representative of three independent experiments. (**d**) Immunoprecipitation–western blot analysis demonstrating the binding of Kif2b and wild-type or mutant CEP170 (**e**) Immunoprecipitation–western blot analysis demonstrating the binding of dynein and wild type or mutant NuMA.

To validate whether inhibition of TBK1-mediated phosphorylation of CEP170 or NuMA is the origin of mitotic errors, we generated phosphorylation site mutant constructs of CEP170 and NuMA by substituting serine residues phosphorylated by TBK1 to alanine and threonine to valine on CEP170 and NuMA. To examine whether the mutants exhibit any changes in function or localization pattern, we transfected CEP170 or NuMA depleted HeLa cells with GFP-tagged wild-type or mutant CEP170 (Fig. 8a, upper panel) or NuMA (Fig. 8a, lower panel) and subsequently immunostained with alpha tubulin antibody. Confocal microscopy revealed that the cells transfected with mutant constructs showed mitotic aberrations like multipolar spindles; the results are quantified in Fig. 8b. We confirmed that TBK1 could not phosphorylate these mutants in kinase assays, using GST tagged wild-type or mutant CEP170 and NuMA in *in vitro* kinase assays as substrates of TBK1. As seen in Fig. 8c, TBK1 could phosphorylate wild-type CEP170 and NuMA, while the mutants showed only marginal levels of phosphorylation, suggesting that the mutated residues were indeed TBK1 targets.

Further, we also assessed whether there is any difference in the interaction of the mutant proteins with Kif2b or Dynein. For this we performed immunoprecipitation–western blot analysis to examine the association of Kif2b and wild-type or mutant CEP170. There was lesser binding of Kif2b to mutant CEP170 as compared with wild-type CEP170 (Fig. 8d). In similar experiments we observed that interaction of Dynein with mutant NuMA was significantly reduced compared to wild-type NuMA and dynein interaction (Fig. 8e). Taken together, these studies show that TBK1-mediated modulation of CEP170 and NuMA facilitates proper mitotic spindle formation and mitosis.

## Discussion

The successful partition of replicated genomes requires chromosome attachment to opposite poles of mitotic spindles or centrosomes during cell division^34^. Any defects in this assembly result in increased frequency of chromosome segregation errors and chromosomal instability, which may accelerate tumour initiation and progression^35^,^36^,^37^,^38^. Kinases direct several of the complex steps of mitosis, including the serine/threonine kinases CDK1, PLK1, NEK2 and the Aurora Kinases^39^,^40^,^41^. Further, microtubule dynamics is controlled by the kinesin 13 family of depolymerases (Kif2a, Kif2b and Kif2c/MCAK) and Kif2b stably associates with the microtubule-binding protein CEP170 that recruits Kif2b to the mitotic spindle^20^,^27^. Our studies now show that TBK1-directed phosphorylation of CEP170 is required for its interaction with Kif2b and with centrosomes, and that inhibition or knockdown of TBK1 augments microtubule stability to inhibit mitosis by disabling the TBK1–CEP170 complex and reducing the interactions of CEP170 with centrosomes. Furthermore, we show that TBK1 phosphorylates and controls the association of NuMA with dynein, and its localization to the spindle poles. NuMA is phosphorylated during the initial stages of mitosis by p34/cdc2 and NuMA phosphorylation regulates its interaction with dynein, which deposits NuMA at spindle poles where it tethers microtubules^15^,^42^. Our studies now reveal TBK1 as another kinase that phosphorylates NuMA and that is required for its association with dynein and for localization of NuMA to the centrosomes in mitotic cells.

Although our study demonstrates that TBK1 is activated before the onset of mitosis and is required for successful cell division, it is not clear what upstream signalling events or kinases initiate mitosis-specific activation of TBK1. Recent studies indicate that clustering of TBK1 molecules allow inter-dimer interactions leading to trans-autophosphorylation^43^. In addition, TBK1 can be activated by IKKβ in immune cells on innate immune signalling^44^. During viral infection, GSK3β is known to associate with TBK1 and promote self-association and autophosphorylation at S172; however, the effect of GSK3β on TBK1 is independent of its kinase activity^45^. Similar mode of mitotic kinase activation is observed in case of Aurora A: LIM protein ajuba interacts with Aurora A in mitotic cells and induces autophosphorylation and consequent activation of Aurora A^46^. It is tempting to speculate that other interacting proteins might be triggering trans-autophosphorylation of TBK1 for mitotic commitment. Further investigations are needed to assess whether canonical IKKs or mitotic kinases such as CDK1 or a non-kinase interacting partner activate TBK1 during mitosis.

TBK1 has been shown to be necessary for the survival of mutant K-Ras-expressing tumour cells, where TBK1 signalling induces c-Rel and BCL-XL^10^ and activates Akt^7^. A recent genome wide RNA interference (RNAi) screen to identify synthetic lethal interactions of mutant K-Ras demonstrated an enrichment for genes the encode proteins having mitotic functions including PLK-1 and anaphase promoting complex/cyclosome^47^. Our finding that TBK1 functions are necessary for mitosis suggests that the dependency of mutant K-Ras tumour cells on TBK1 for cellular transformation also reflects its roles in directing progression through mitosis, by targeting PLK1 as well as other targets like CEP170 and NuMA. The mitotic roles of TBK1 also has implications for the innate immune response, where TBK1 mediates signalling events downstream of TLRs and plays a pivotal role in the activation of innate immunity in response to viral and bacterial infections^3^,^48^. Surprisingly, recent studies suggest that TLRs are also expressed in several cancer types and that TLR signalling contributes to tumorigenesis by promoting cell survival, chemoresistance, immune evasion and metastasis^43^,^49^. Thus, mitotic functions of TBK1 are likely to play an essential role in such oncogenic processes. Finally, this new role of TBK1 also opens new avenues for targeting TBK1 in cancer, to induce mitotic catastrophe and cell death.

## Methods

### Cell lines and reagents

A549 (mutant K-Ras) NSCLC cells were cultured in F12K medium with 10% serum (Cellgro). H1650, Calu-6, PC9 (wild-type K-Ras) and H460 (large cell; wild-type K-Ras) human NSCLC cells were grown in RPMI with 10% serum. These cell lines were purchased from ATCC. AALE cells (immortalized human tracheobronchial epithelial cells)^50^, were maintained in BEGM media with growth supplements. To synchronize cells in G1/S, cells were subjected to double-thymidine block (2 mM) or aphidicolin treatment (5 μg ml^−1^) using standard protocols^51^,^52^ and then released with complete media. To inhibit TBK1, cells were treated with 0.5 μM BX795 (Axon Medchem or Tocris Bioscience). GST-NuMA (pGEX6P1-NuMA-C-WT) was provided by Dr Fumiko Toyoshima, Kyoto University, Japan^53^. His6-tagged CEP170 was a kind gift from Dr Eric Nigg, University of Basel, Switzerland^14^. PLK1 inhibitor BI2536 (Selleck chemicals) was used at 100 nM concentration for inhibiting PLK1 kinase activity.

### Lysate preparation and western blots

Lysates from cells synchronized at G1/S or released with complete media to enter mitosis, or those treated with BX795, were prepared by Nonidet P-40 (Igepal CA-630; NP-40) lysis as described^54^ and 100 μg protein was run on polyacrylamide-SDS gels and immunoblotted with the indicated antibodies. Phospho (S172) TBK1 (#5483), phospho histone H3 (Abcam #5176) and total TBK1 antibody was purchased from Cell Signaling, and antibodies to CEP170 (Invitrogen or Abcam), NuMA (BD #610561) or Abcam#109262), alpha tubulin (Sigma #T5168 or Abcam #15246) were used in both western blots and immunofluorescence. Monoclonal antibody to actin was purchased from Sigma Chemical Co. (#A1978, clone AC-15). All primary antibodies were used at 1:1,000 dilution for western blots, except actin antibody, which was used at 1:50,000 dilution.

Interaction between proteins *in vivo* was analysed by immunoprecipitation–western blot analyses with 250 μg of lysate and 1 μg of the indicated antibody as described^54^. Polyclonal TBK1 antibody was obtained from Cell Signaling (#2013). Western blot images presented in the article are representative of at least two independent experiments. Full scans of the important images are provided as Supplementary Fig. 10.

### Detection of mitotic and 4*n*+ population of cells using flow cytometry

Identification and quantification of mitotic and 4*n*+ population of cells was based on detection of histone H3 phosphorylated at Serine 10 (H3pS10, mitotic marker-Abcam) along with differential staining of cellular DNA by DAPI using flow cytometry using standard protocols^55^. Briefly, asynchronously growing control AALE or H460 cells, as well as BX795-treated cells, were trypsinized and fixed in 1% formaldehyde, permeabilized with 70% ethanol and immunostained with pH3S10 antibody (Abcam) followed by Alexa Fluor-488 conjugated secondary antibody and DNA was counterstained by DAPI. Bivariate analysis of cellular green fluorescence intensity (Alexa Fluor-488 used as secondary) versus fluorescence from DAPI allowed to distinguish G0/G1, S, G2 and M populations. Flow cytometry was performed using LSRII flow cytometry system. Similar protocols were followed for the identification and quantification of mitotic and 4*n*+ population of cells after cell synchronization by double-thymidine block and subsequent release of the cells to mitotic phase±the TBK1 inhibitor BX795.

### siRNA transfections

siRNA specifically targeting TBK1 as well as a non-targeting control siRNA was purchased from Santa Cruz biotechnology (siRNA#1) or Ambion (siRNA#2). siRNAs were transfected using Oligofectamine (Invitrogen Corporation) according to manufacturer's protocols.

All data were graphically represented and statistically analysed using Microsoft Office Excel 2003 (Microsoft Corporation, Redmond, WA). In all analyses, means and 95% confidence intervals were estimated. Statistical analysis was performed using Student *t*-test and values were considered significant when the *P* value was <0.05.

### Lentiviral shRNA production and infection

Lentiviral vectors expressing shRNAs specific for control sequences or for TBK1 were obtained from the TRC shRNA library. Lentiviruses were produced by transfection of 293FT cells with vectors encoding control or TBK1 shRNA (1 μg) together with the packaging plasmids using Fugene HD (Roche). Culture supernatants containing lentivirus were collected 48 and 72 h after transfection. Virus was pooled and stored at −80 °C. Cells were infected using the supernatant containing virus in polybrene containing media.

### Immunofluorescence and confocal microscopy

A549, H1650, H460, Calu-6, PC9 or AALE cells were plated onto poly-D-lysine-coated eight-well glass chamber slides (5,000 cells per well) for immunostaining. The cells were fixed with 10% buffered formalin and indirect immunofluorescence was performed as described^56^. Primary antibodies used were phospho-TBK1 (Cell signaling #5483S) or CEP170 mouse monoclonal-72-413-1 (Invitrogen #41-3200) at 1:100 dilution or 1:500 dilution of rabbit monoclonal NuMA (EP3976 Abcam #109262), Alpha tubulin (1:2,000, Sigma #T6074), Dynein intermediate chain (Abcam#ab23905) and Kif2b (Abcam #ab98214 or Novus #NBP86002), γ Tubulin (Santa Cruz #sc51715) were used at 1:100 dilution. Anti-rabbit Alexa Fluor-488 or anti- mouse Alexa Fluor-594 (Molecular Probes) was used as secondary antibody. DAPI (Vector Labs) was used to stain the nuclei. Cells were visualized with a DM16000 inverted Leica TCS SP5 tandem scanning confocal microscope with a × 63/1.40NA oil immersion objective. Images and Z-stacks were produced with three cooled photomultiplier detectors and analysed with the LAS AF software version 1.6.0 build 1016 (Leica Microsystems, Germany).

### TBK1 expression constructs

Human *TBK1* was PCR amplified using the following primer pairs 5′-TBK1-BamF1-ATCGTGG ATCCAGATGCAGAGCACTTCTAATCATC-3′ and 5′-TBK1-XhoR1-GTCAGCTCGAGCTAAAGACAG TCAACGTTGCGA-3′, and then cloned into pGEX5X1 to express TBK1 as GST fusion protein in bacteria. For *in vitro* transcription and translation, TBK1 was cloned into pcDNA3 using the following primer pair:—5′-TBK1-KZK-F2-CCATGGAGATGCAGAGCACTTCTAATCATC-3′ and 5′-TBK1-KZK-R2-CTAAAGACAGTCAACGTTGCGAAGGCCAC-3′.

### *In vitro* kinase assays

For kinase assays, enzymatically active TBK1 was purchased from Invitrogen/Life Technologies. The kinase reaction was carried out in a reaction buffer (50 mM HEPES, pH 7.9, 10 mM MgCl_2_, 5 mM MnCl_2_, 1 mM DTT, and 10 mM β-glycerophosphate) containing GST-NuMA or His-CEP170 as substrate, 100 μM ATP, and 10 μCi γ^32^P-ATP along with 100 ng TBK1 at 37 °C for 30 min^54^,^57^. Histone H1 was used as positive control and the kinase reaction was performed at 30 °C for 30 min. Samples were then boiled in SDS sample loading buffer and resolved by polyacrylamide gel electrophoresis and dried using a vacuum gel dryer. The phosphorylation of NuMA, CEP170 or Histone H1 was visualized by autoradiography. Subsequently, the gel was rehydrated and stained with Coomassie Brilliant Blue to confirm the levels of substrate in each reaction. Images of kinase assay results presented in the article are representative of at least two independent experiments.

### *In vitro* GST-binding assays

GST, GST-TBK1, GST-NuMA and His-tagged CEP170 were expressed in bacteria and were purified as bound to glutathione-Sepharose beads or His beads as described^54^. Beads were washed three times with PBS, and protein integrity was checked by polyacrylamide gel electrophoresis and Coomassie Blue staining. ^35^S-Methionine-labelled lysates of TBK1 or CEP170 were generated using the rabbit reticulocyte translation system according to the manufacturer's instructions (Promega). Eight microlitres of labelled CEP170 lysate was incubated with an equivalent amount of GST or GST-TBK1 or 8 μl of labelled TBK1 was incubated with GST-NuMA or His- CEP170 beads in a buffer containing 20 mM Tris-HCl (pH 7.5), 0.5% Nonidet P-40 (Igepal CA-630), 50 mM KCI, 500 mM EDTA, and 3 mg ml^−1^ bovine serum albumin, 1 mM dithiothreitol and 0.5 mM phenylmethylsulfonyl fluoride^57^. Samples were incubated for 2 h at 4 °C and then washed in binding buffer six times. Bound proteins were eluted in gel loading buffer and resolved by SDS-PAGE gel electrophoresis. Gels were dried using a vacuum gel dryer and the signal from the gels were obtained by autoradiography.

### Identification of CEP170 amino-acid residues phosphorylated by TBK1 by mass spectrometry

*In vitro* kinase reactions were performed using CEP170 as substrate +/− enzymatically active TBK1 as noted above to identify amino acids specifically phosphorylated by TBK1. Samples were subsequently boiled in SDS sample-loading buffer and resolved by polyacrylamide gel electrophoresis. To reduce and alklylate proteins, destained gel slices were treated with TCEP and iodoacetamide. Following in-gel digestion, using trypsin, peptides were extracted and concentrated under vacuum centrifugation. A nanoflow ultra high-performance liquid chromatograph (RSLC, Dionex) coupled to an electrospray ion-trap mass spectrometer (LTQ-Orbitrap, ThermoScientific) was used for tandem mass spectrometry peptide sequencing experiments. The sample was first loaded onto a precolumn (2 × 75 μm ID packed with C18 reversed-phase resin, 5 μm, 100 Å) and washed for 8 min with aqueous 2% acetonitrile and 0.04% trifluoroacetic acid. The trapped peptides were eluted onto the analytical column, (C18, 75 μm ID × 50 cm, Pepmap 100, Dionex). The 120-min gradient was programmed as follows: 95% solvent A (2% acetonitrile+0.1% formic acid) for 8 min, solvent B (90% acetonitrile+0.1% formic acid) from 5 to 15% for 5 min, 15 to 40% for 85 min, then solvent B from 50 to 90% B for 7 min and held at 90% for 5 min, followed by solvent B from 90 to 5% for 1 min and then re-equilibration for 10 min. The flow rate on analytical column was 300 ml min^−1^. Five tandem mass spectra were collected in a data-dependent manner following each survey scan. The MS scans were performed in Orbitrap to obtain accurate peptide mass measurement and the MS/MS scans were performed in linear ion trap using 60-s exclusion for previously sampled peptide peaks. Sequest and Mascot searches were performed against the Swiss-Prot human database^58^,^59^. Two trypsin-missed cleavages were allowed, the precursor mass tolerance was 1.08 Da. MS/MS mass tolerance was 0.8 Da. Dynamic modifications included carbamidomethylation (Cys), oxidation (Met) and phosphorylation (Ser/Thr/Tyr).

### Microtubule stability assays

A549, H1650 or H460 cells were plated in eight-well chamber slides at a density of 5,000 cells per well and treated with shcontrol or shTBK1 lentiviral supernatants 24 h after seeding. Cells were grown for 48 h and were incubated in growth media with 0.1 μM nocodazole for 15 min for depolymerizing microtubules. Cells were fixed with formalin and double immunofluorescence assays were performed as described^23^,^24^.

Microtubule depolymerization by cold treatment and regrowth or reassembly was conducted by incubating the cells on ice for 45 min. Subsequently, the cells were washed with prewarmed media and incubated in warm media for 7 min at 37 °C Cells were fixed in formalin and stained for alpha tubulin and CEP170 (ref. 25).

To assess microtubule stability in TBK1 overexpressing cells, A549-GFP control cells and A549 cells overexpressing TBK1 (A549-TBK1) were grown on chamber slides at a density of 5,000 cells per well for 48 h. Cells were then incubated in growth media with 5 μM nocodazole for 1 h, washed with prewarmed media, and incubated in prewarmed media without Nocodazole for 7 min. Cells were fixed with formalin and double immunofluorescence assays were performed as indicated above^26^.

### Proximity ligation assay

Proximity ligation assays were performed using the Duolink system^22^ (Sigma). After treating H460 cells with 5 μM Scrambled peptide conjugated with penetratin (SCR-Pen) or CEP peptide (CEP-Pen) for 24 h, cells were fixed with 10% buffered formalin. Cells were permeabilized with 0.5% Triton X-100 in PBS for 10 min and blocked with 5% Normal goat serum for 1 h in a humidity chamber. The two primary antibodies prepared in 5% normal goat serum solution to appropriate concentrations (3:100 of each antibody; TBK1 from Cell Signaling and CEP170 from Invitrogen) was added to the slide and allowed to incubate at 4 °C overnight in a humidity chamber. After incubation the chamber slides were washed twice with wash buffer A containing 0.15 M NaCl, 0.02 M Tris, 0.05% Tween 20, pH 7.4 for 5 min. Plus and minus PLA probes (secondary antibody conjugated to a unique oligonucleotide; plus for rabbit and minus for mouse) were added to the slide and incubated for 1 h. After incubation, the slides were washed twice with wash buffer A for 5 min. The two PLA probes were joined to a closed circle (if they are in close proximity; <40 nm) by incubating in a ligation solution containing two oligonucleotides and a ligase in humidity chamber at 37 °C for 30 min. After incubation, the cells were washed two times for 2 min in wash buffer A in a Coplin jar with gentle agitation. Amplification was done by incubating in amplification solution consisting of nucleotides and fluorescently labelled oligonucleotides (detection probes; green) along with a polymerase and added directly onto cells and incubated in humidity chamber at 37 °C for 120 min. A rolling circle amplification reaction was performed to generate concatameric DNA onto which detection probes subsequently hybridize. After incubation, the cells were washed twice in wash buffer B containing 0.1 M NaCl, 4.24 g Tris base, 0.2 M Tris (pH 7.5) for 10 min. Then slides were washed for 1 min with 0.01% wash buffer B in water. The slides were allowed to dry in the dark for 15 min and then mounted with cover slip using mounting medium with DAPI (Vectashield). Images were acquired with the LAS AF software version 1.6.0 build 1016 (Leica Microsystems, Germany). For quantification of green fluorescent spots representing points of localization Definiens Developer 1.5 (Definiens AG, Munich, Germany) was used. All the cells within four images were analysed for each treatment. Data shown in Fig. 8f represents the average number of foci per cell from these images.

### Live cell imaging

A microscope outfitted with XL S1 full enclosure incubator (37 °C, 5% CO_2_, and humidity) was used for live cell imaging of control H460 or HeLa cells and those treated with TBK1 inhibitors BX795 (0.5 μM) and MRT (2 μM). HeLa cells stably transfected with GFP-tagged alpha tubulin and mCherry-tagged H2B were a kind gift from Drs Daniel Wolfram Gerlich (IMBA, Austria) and Buzz Baum (LMCB, UCL). We used the cell line from Dr Gerlich's lab for all the live cell imaging shown in supplementary movies. Phase contrast and fluorescence images were captured at 5 min intervals for 48 h using the AxioCam MRm3 CCD camera through a × 20 0.5 NA Plan Apochromat objective with GFP and DSRED filter sets for imaging HeLa cells transfected with GFP-Alpha Tubulin and mCherry-H2B. Phase contrast images of H460 cells were captured at 5-min intervals for 48 h using the AxioCam MRm3 CCD camera and a × 20 0.5NA Plan Apochromat objective. Axiovision version 4.8 software suite was used to acquire and process images. Definite focus was used stabilize focal plane during the acquisition (Carl Zeiss Inc., Germany).

### Centrosome isolation

Centrosomes from A549 and H460 non-small cell lung cancer cell lines were isolated following standard protocols (discontinuous sucrose gradient fractionation for centrosome isolation^16^,^17^, and subsequently subjected to western blot analysis. The cells were collected for centrosome isolation after brief treatment with nocodazole and cytochalasin D to disrupt microtubules and actin cytoskeleton.

### Generation of phosphorylation site mutants

C terminus of CEP170-containing 2,331 nucleotides (777 amino acids) was synthesized (Genescript) with all serine and threonine residues targeted by TBK1 mutated to alanine or valine, respectively (see Supplementary Tables 1 and 2 for the residues; additional residues identified by SILAC experiments were also mutated). This includes the following residues: S865A, S866A, S881A, S887A, T891V, S930A, S933A, S939A, S942A, S1123A, S1278A, S1362A, S1529A, S1560A, S1565A. Similarly, NuMA C terminus containing 1,248 nucleotides (416 amino acids) was synthesized with serine and threonine residues targeted by TBK1 mutated to alanine or valine respectively (S1728A, S1772A, S1872A, T1906V, S1969A, S1991A, S2051A, T2055V, S2062A). The mutated C terminus and the corresponding wild-type C terminus of both CEP170 and NuMA were cloned into PGEX-5X-1 or pEGFP-C1 to express as GST or GFP-tagged protein respectively.

## Additional information

**How to cite this article:** Pillai, S. *et al*. Tank binding kinase 1 is a centrosome-associated kinase necessary for microtubule dynamics and mitosis. *Nat. Commun*. 6:10072 doi: 10.1038/ncomms10072 (2015).

## Supplementary Material

Supplementary Figures and Supplementary Tables Supplementary Figures 1-10 and Supplementary Tables 1-2

Supplementary Movie 1 H460-Control. Live cell imaging of H460 control cells using the AxioCam MRm3 CCD camera through a 20x 0.5NA Plan Apochromat objective. Phase contrast images were captured at 5 minute intervals for 48 hours. Axiovision version 4.8 software suite was used to process images to make representative shorter video clips.

Supplementary Movie 2 H460-BX795. Live cell imaging of H460 cells treated with 0.5μM of TBK1 inhibitor BX795 using the AxioCam MRm3 CCD camera through a 20x 0.5NA Plan Apochromat objective. Phase contrast images were captured at 5 minute intervals for 48 hours. Axiovision version 4.8 software suite was used to process images to make representative shorter video clips.

Supplementary Movie 3 H460-MRT. Live cell imaging of H460 cells treated with 5μM of TBK1 inhibitor MRT67307 using the AxioCam MRm3 CCD camera through a 20x 0.5NA Plan Apochromat objective. Phase contrast images were captured at 5 minute intervals for 48 hours. Axiovision version 4.8 software suite was used to process images to make representative shorter video clips.

Supplementary Movie 4 HeLa Control. Live cell imaging of HeLa cells transfected with GFP-a-tubulin and mCherry-H2B using the AxioCam MRm3 CCD camera through a 20x 0.5NA Plan Apochromat objective with GFP and DsRED filter sets. Phase contrast and fluorescent images were captured at 5 minute intervals for 48 hours. Axiovision version 4.8 software suite was used to process images to make representative shorter video clips.

Supplementary Movie 5 HeLa MRT. Live cell imaging of HeLa cells transfected with GFP-a-tubulin and mCherry-H2B after treating with 2μM of TBK1 inhibitor MRT67307 using the AxioCam MRm3 CCD camera through a 20x 0.5NA Plan Apochromat objective with GFP and DsRED filter sets. Phase contrast and fluorescent images were captured at 5 minute intervals for 48 hours. Axiovision version 4.8 software suite was used to process images to make representative shorter video clips.

Supplementary Movie 6 HeLa BX795. Live cell imaging of HeLa cells transfected with GFP-α-tubulin and mCherry-H2B after treating with 0.5μM of TBK1 inhibitor BX795 using the AxioCam MRm3 CCD camera through a 20x 0.5NA Plan Apochromat objective with GFP and DsRED filter sets. Phase contrast and fluorescent images were captured at 5 minute intervals for 48 hours. Axiovision version 4.8 software suite was used to process images to make representative shorter video clips.
