# Supplementary material for: Tank binding kinase 1 is a centrosome-associated kinase necessary for microtubule dynamics and mitosis
Source: Nat Commun. 2015 Dec 10;6:10072. doi: 10.1038/ncomms10072 (PMC4682058; doi:10.1038/ncomms10072)
Supplement: Supplementary Figures and Supplementary Tables — Supplementary Figures 1-10 and Supplementary Tables 1-2 [file ncomms10072-s1.pdf]

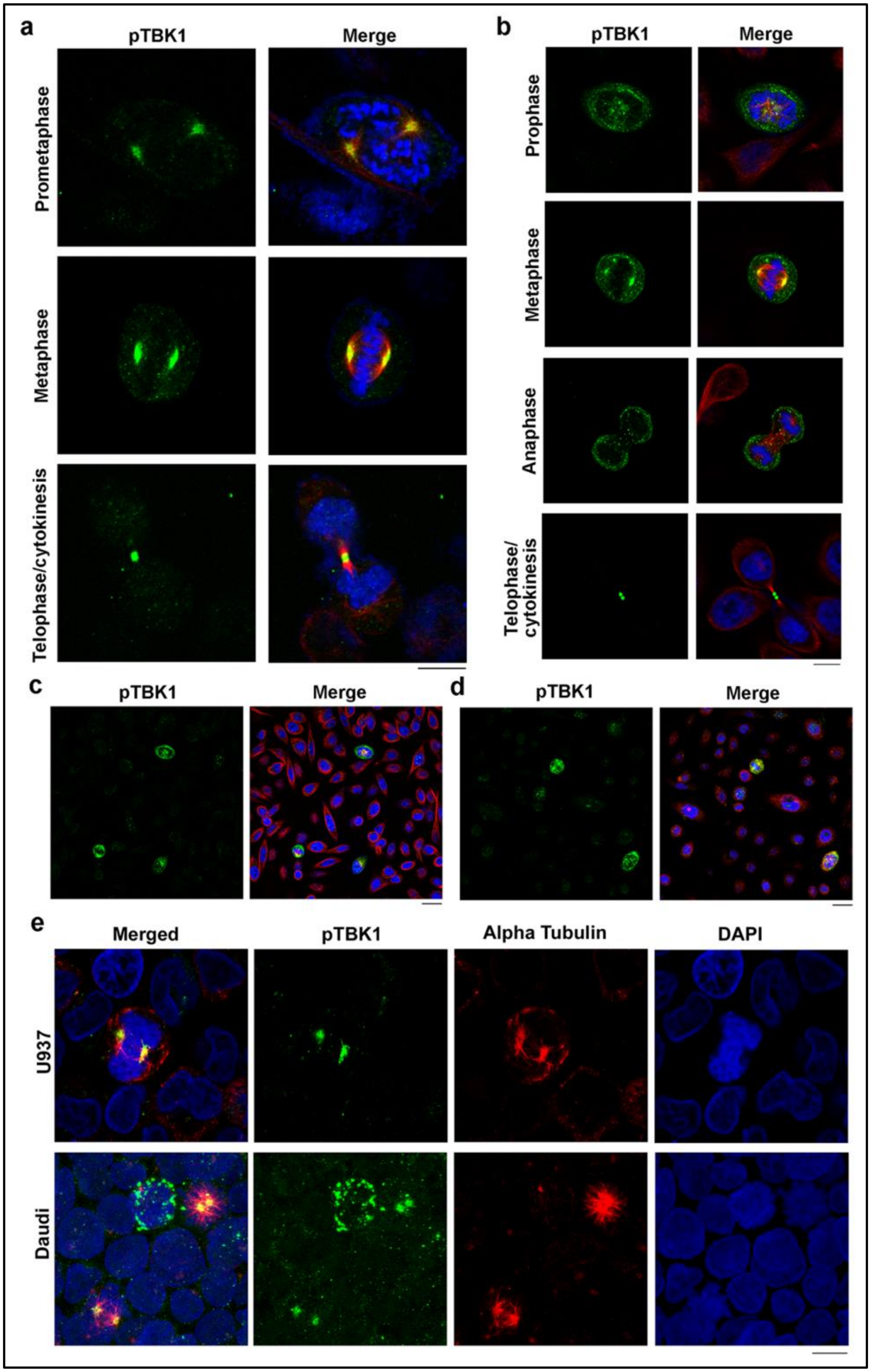
a
pTBK1
Merge
Prometaphase
Metaphase
Telophase/cytokinesis
b
pTBK1
Merge
Prophase
Metaphase
Anaphase
Telophase/
cytokinesis
c
pTBK1
Merge
d
pTBK1
Merge
e
Merged
pTBK1
Alpha Tubulin
DAPI
U937
Daudi

Supplementary Figure 1. Phospho-TBK1 localizes to centrosomes and mitotic spindles non-small cell lung cancer cell lines as well as immune cells during mitosis. (a) Confocal microscopy images of Calu-6 cells at prometaphase, metaphase and telophase/cytokinesis stained for phospho-TBK1 (green), alpha tubulin (red) and DNA (DAPI, blue), scale bar=10 µm

(b) Confocal microscopy images of PC9 cells at prophase, metaphase, metaphase, telophase/cytokinesis and non-mitotic cells stained for phospho-TBK1 (green), alpha tubulin (red) and DNA (DAPI, blue), scale bar=10 µm. (c) Confocal image taken at a lower magnification showing the levels of phospho-TBK1 in mitotic as well as non-mitotic PC9 cells stained for phospho-TBK1 and alpha tubulin scale bar=25 µm. (d) AALE cells stained for phospho-TBK1 and CEP170 scale bar=25µm.

(e) U937 (histiocytic lymphoma cells) and Daudi (Burkitt's lymphoma; B lymphoblast) cells stained for phospho-TBK1 (green), alpha tubulin (red) and DNA (DAPI, blue), scale bar=10 µm.

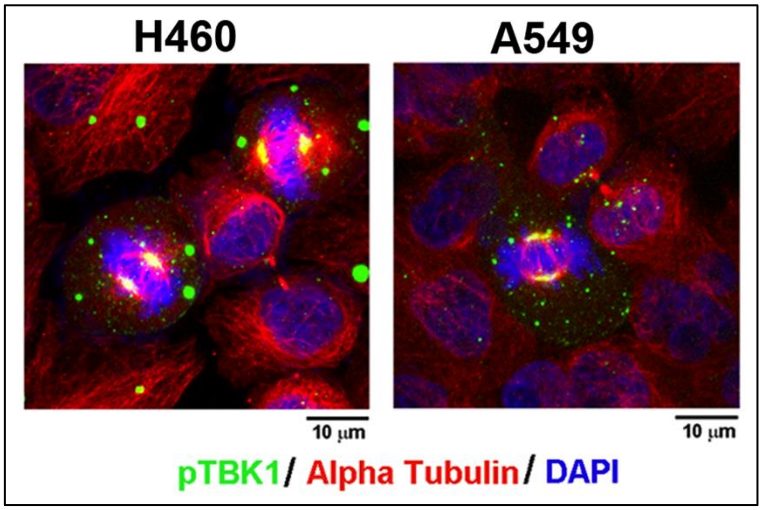

Supplementary Figure 2. Centrosomal localization of phospho-TBK1 as detected by a second antibody

Detection of pTBK1 by immunofluorescence using a second pTBK1 antibody from a different source (Origene) yielded similar results. Phospho-TBK1 (green), alpha tubulin (red) and DNA (DAPI, Blue). This experiment confirms that the centrosomal localization of phosphor-TBK1 can be detected using multiple antibodies from different sources.

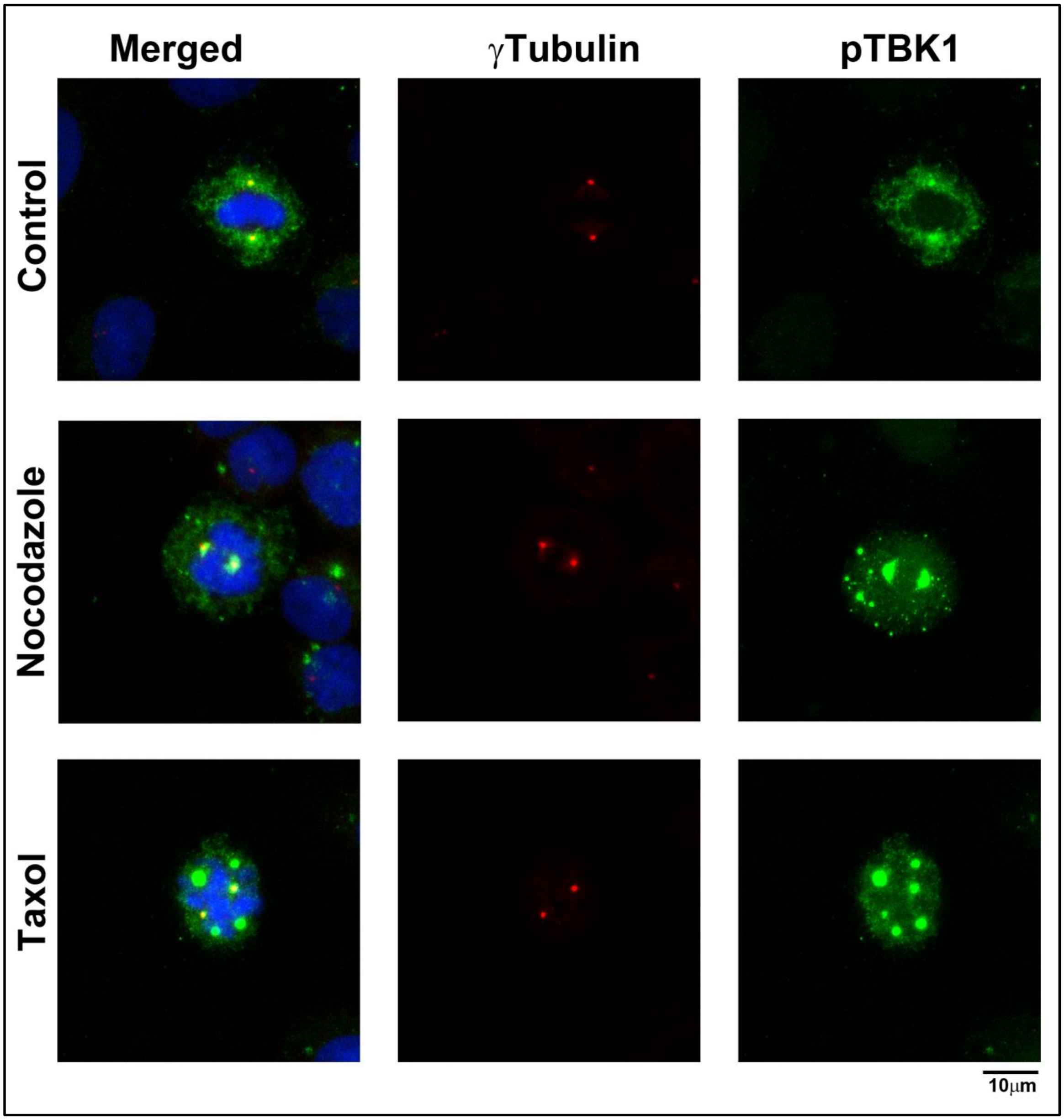

Supplementary Figure 3. Centrosomal localization of TBK1 does not depend on microtubule integrity

Confocal microscopy images of H460 cells treated with nocodazole or taxol demonstrating that pTBK1 localizes to centrosomes when MTs are hyperstabilized by taxol or depolymerized by nocodazole.

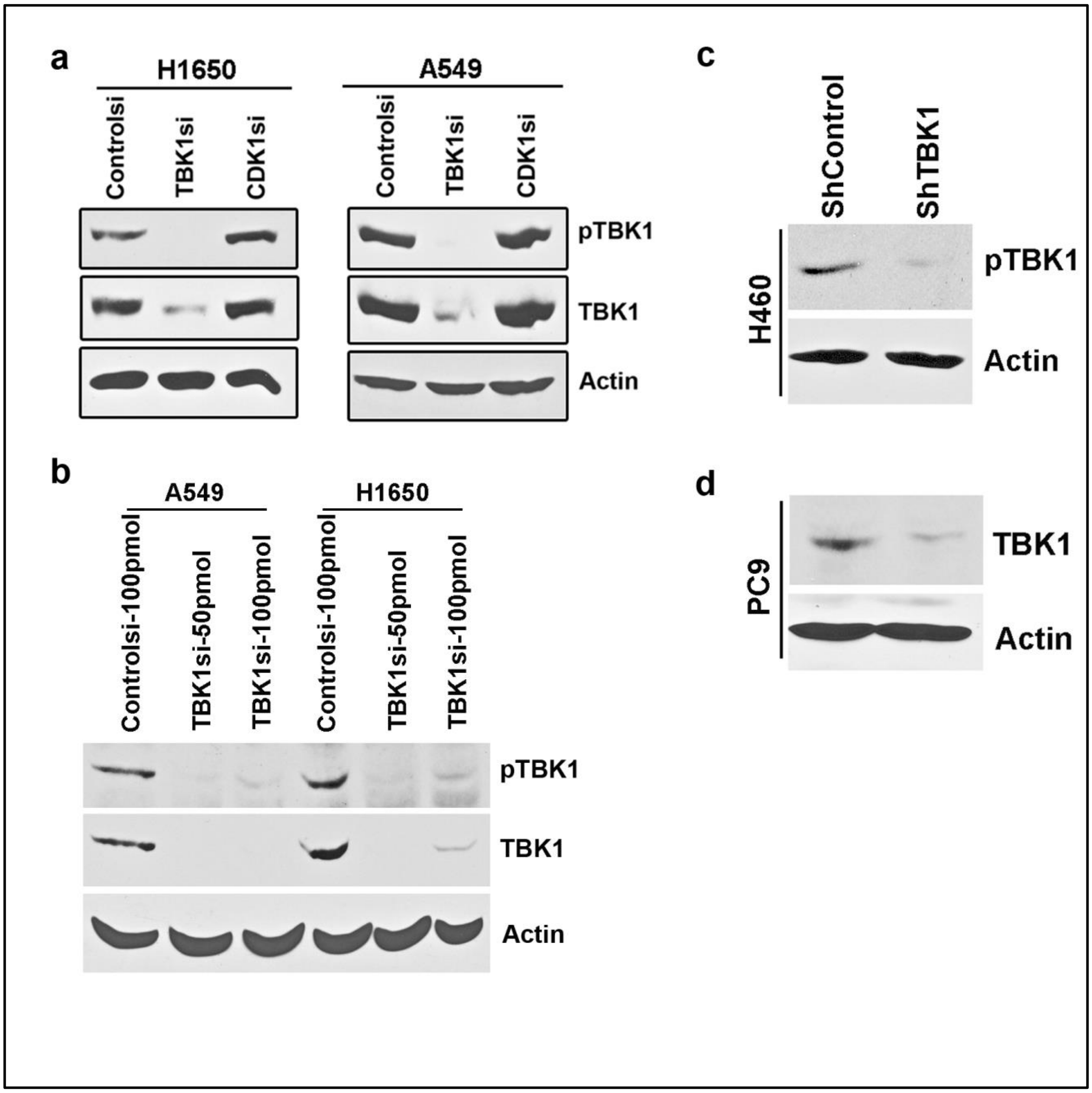

Supplementary Figure 4. Depletion of TBK1 by siRNA and shRNA

(a & b) Western blots showing the levels of phospho-TBK1, TBK1 and after transfection with two different siRNA sequence targeting TBK1 (a) siRNA from SantaCruz (b) siRNA from Ambion. (c & d), Western blots showing the depletion of TBK1 after transducing with shTBK1 lentiviral construct in H460 and PC9.

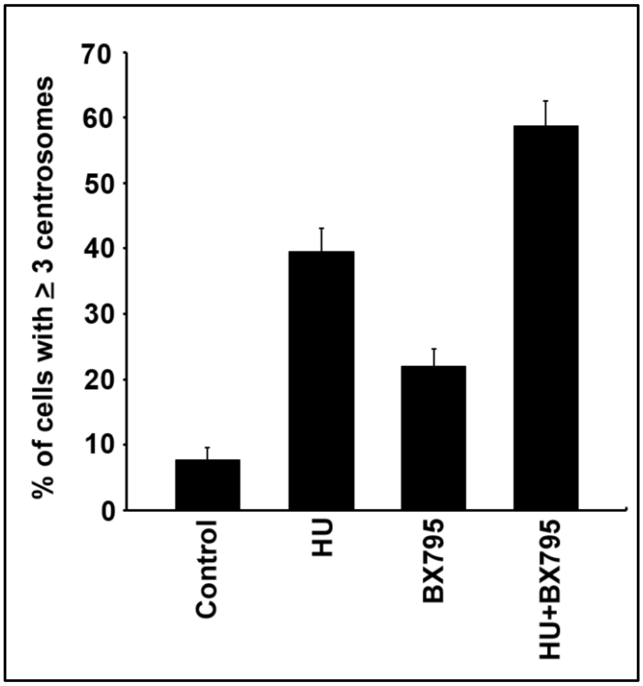

Supplementary Figure 5. TBK1 inhibition increases number of centrosomes

TBK1 inhibition in hydroxyurea (HU) treated U2OS cells using BX795 induced centrosome amplification. TBK1 inhibition alone was sufficient to induce centrosome amplification in 22% of cells; the possibility exists that this is due to incomplete cell division.

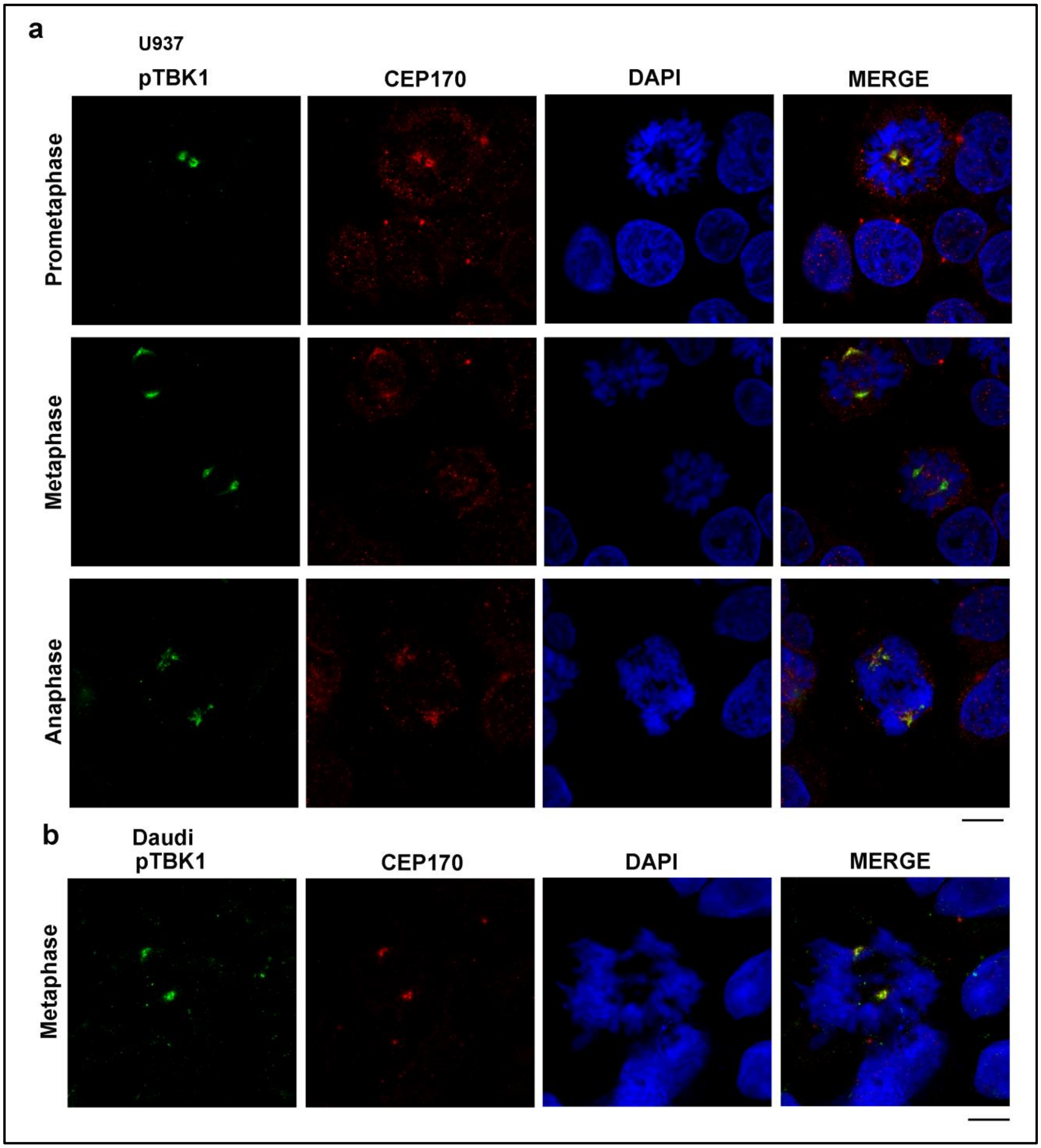

Supplementary Figure 6. Colocalization of phospho-TBK1 with CEP170 in immune cells.

(a,b) Confocal image showing the co-localization of phospho-TBK1 (green) and CEP170 (red) in U937 (histiocytic lymphoma cells) and Daudi (Burkitt's lymphoma; B lymphoblast) cells. Scale bar=10 µm.

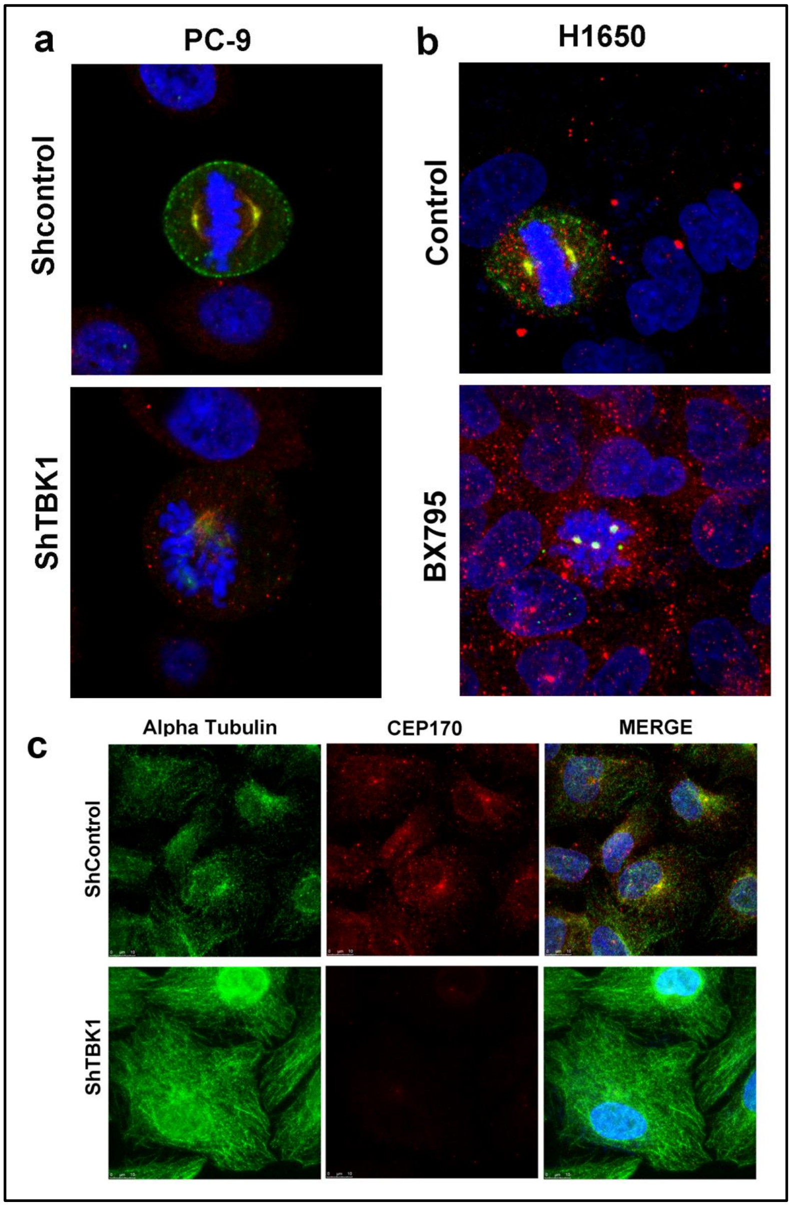

Supplementary Figure 7. Inhibition of TBK1 results in mitotic defects.

(a, b) Mitotic defects observed after depletion of TBK1 in PC9 (a) and inhibition of TBK1 by BX795 in H1650 (b). phospho-TBK1 (green), CEP170 (red) and DAPI (blue). Scale bar=10 µm. (c) Depletion of TBK1 results in inhibition of CEP170 localization to the centrosomes in non-mitotic cells. Microtubules are visualized by staining for Alpha Tubulin (green), CEP170 (red) DNA (DAPI, Blue).

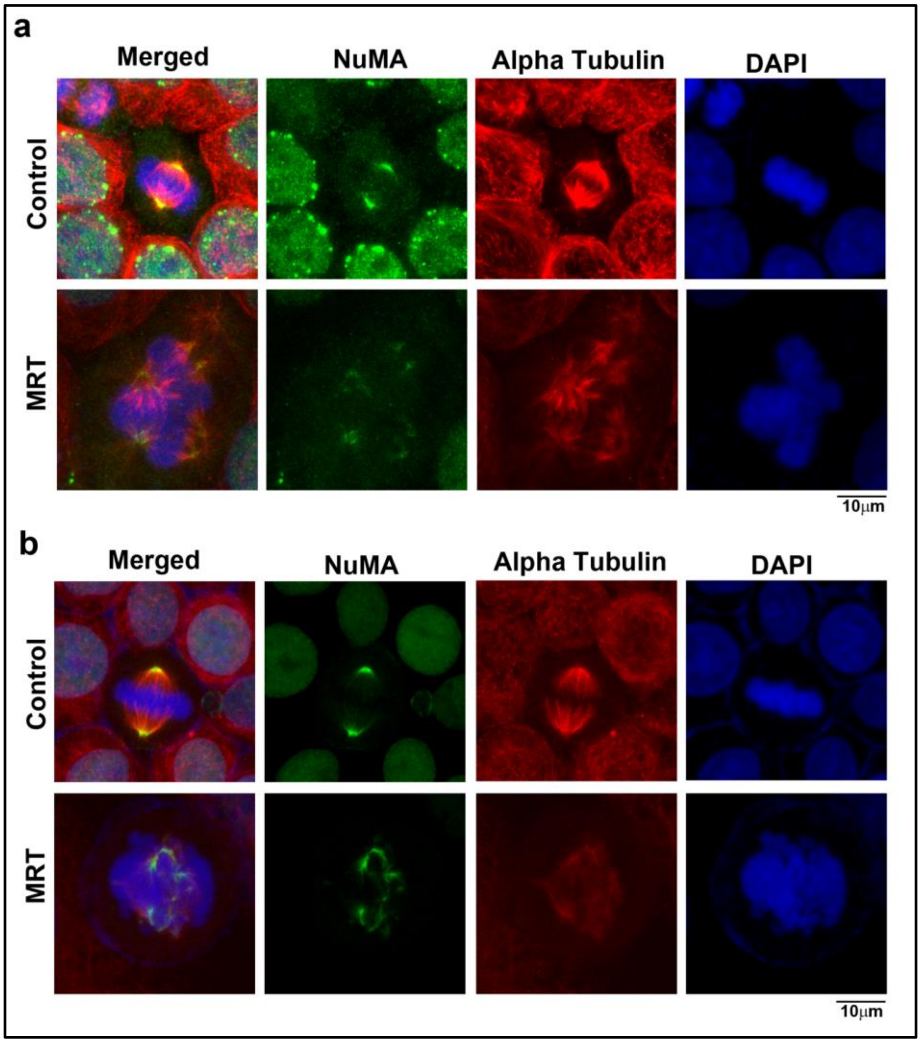

Supplementary Figure 8. TBK1 inhibition affects spindle pole localization of NuMA.

Inhibition of TBK1 by 2 μM MRT affected spindle pole localization of NuMA. H460 (a) and HeLa (b) cells were stained for NuMA (green), Alpha Tubulin (red), DAPI (Blue). Scale bar 10 μm.

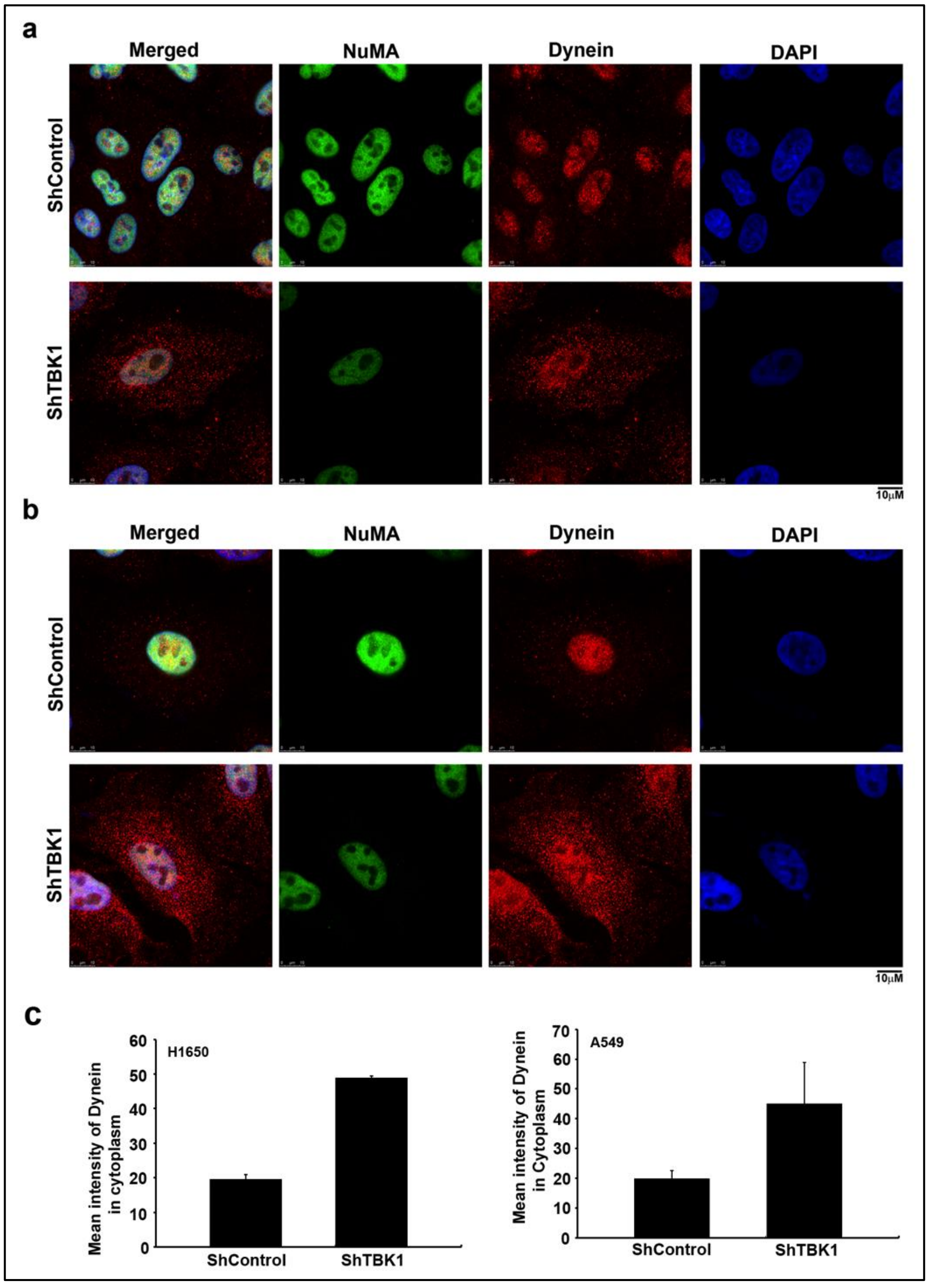

Supplementary Figure 9. TBK1 depletion affects dynein levels in cytoplasm. Immunofluorescence staining for Dynein intermediate chain (red) and NuMA (green) in shcontrol and shTBK1 transfected H1650 (a) and A549 (b) cells. Scale bar 10 µm. (c) Quantification of signal from immunofluorescence images showing alteration in Dynein intermediate chain levels in the cytoplasm after depletion of TBK1.

Supplementary Figure 10. Uncropped images of western blots and gels.

The full scans of the important western blots and gels used in Figures 1, 2, 5, 6, 7 and 8

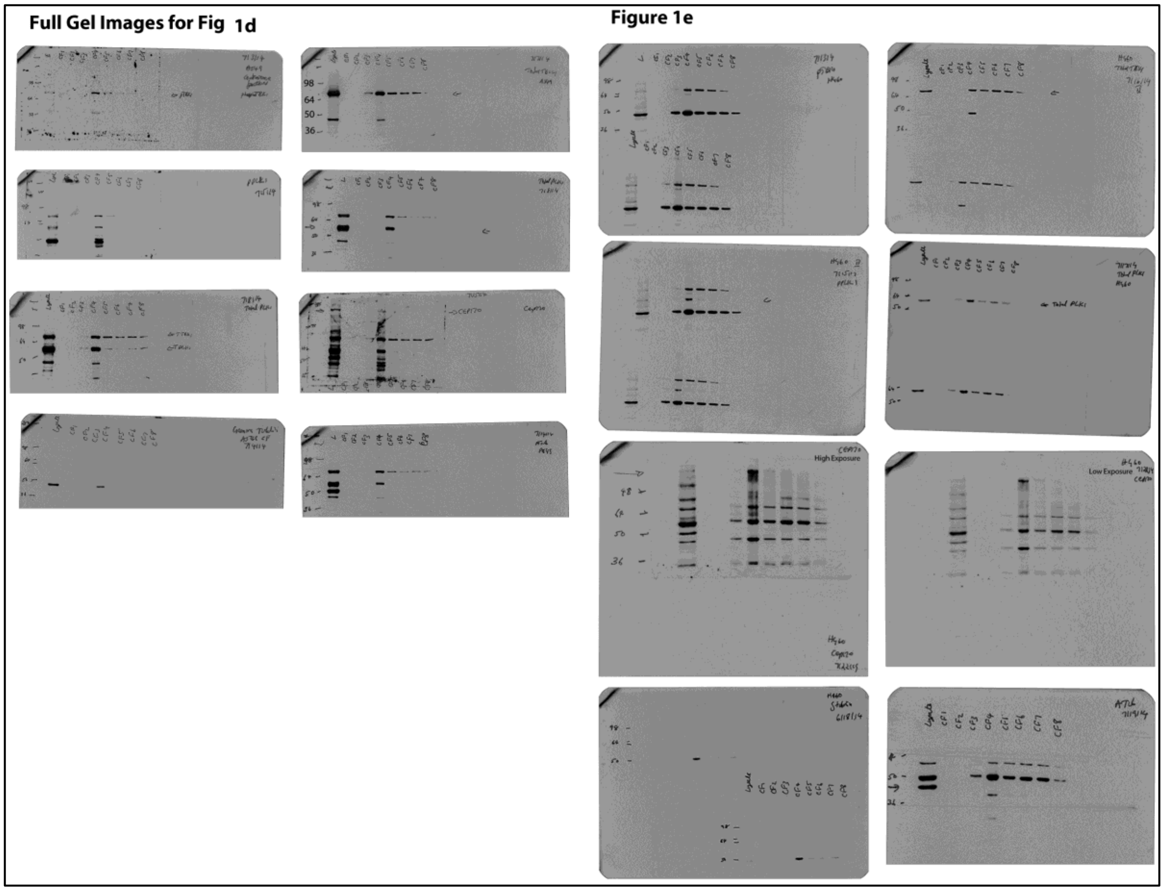

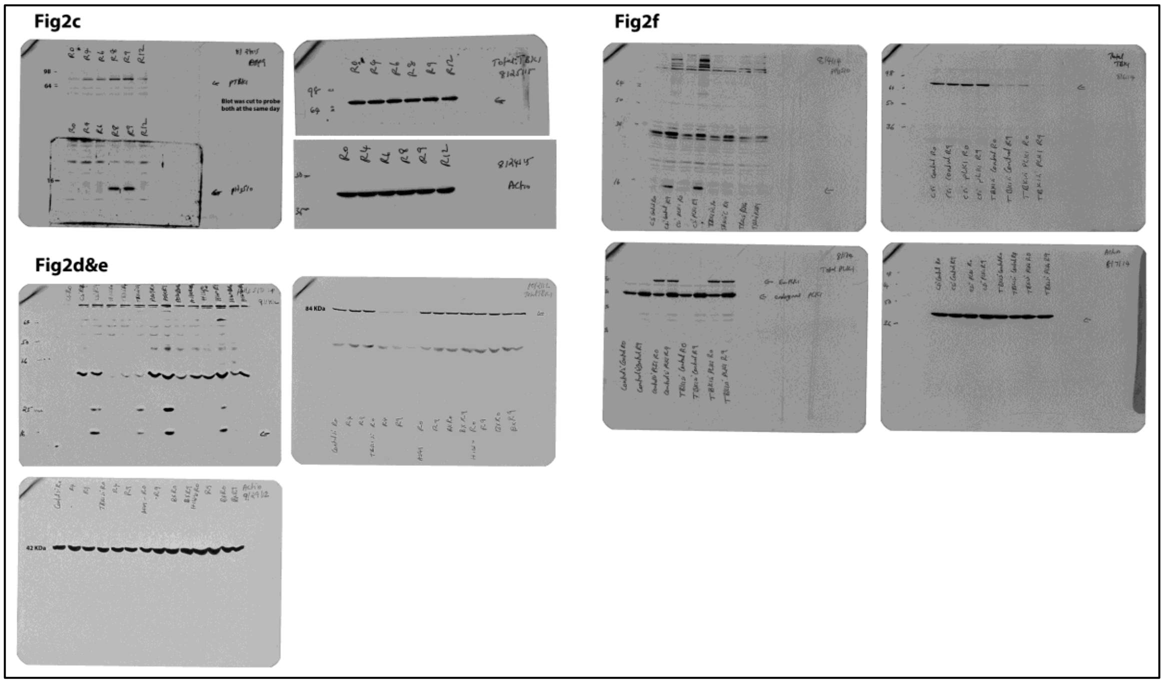
Fig2c
Blot was cut to probe both at the same day
Fig2d&e
84 KDa
42 KDa
Fig2f

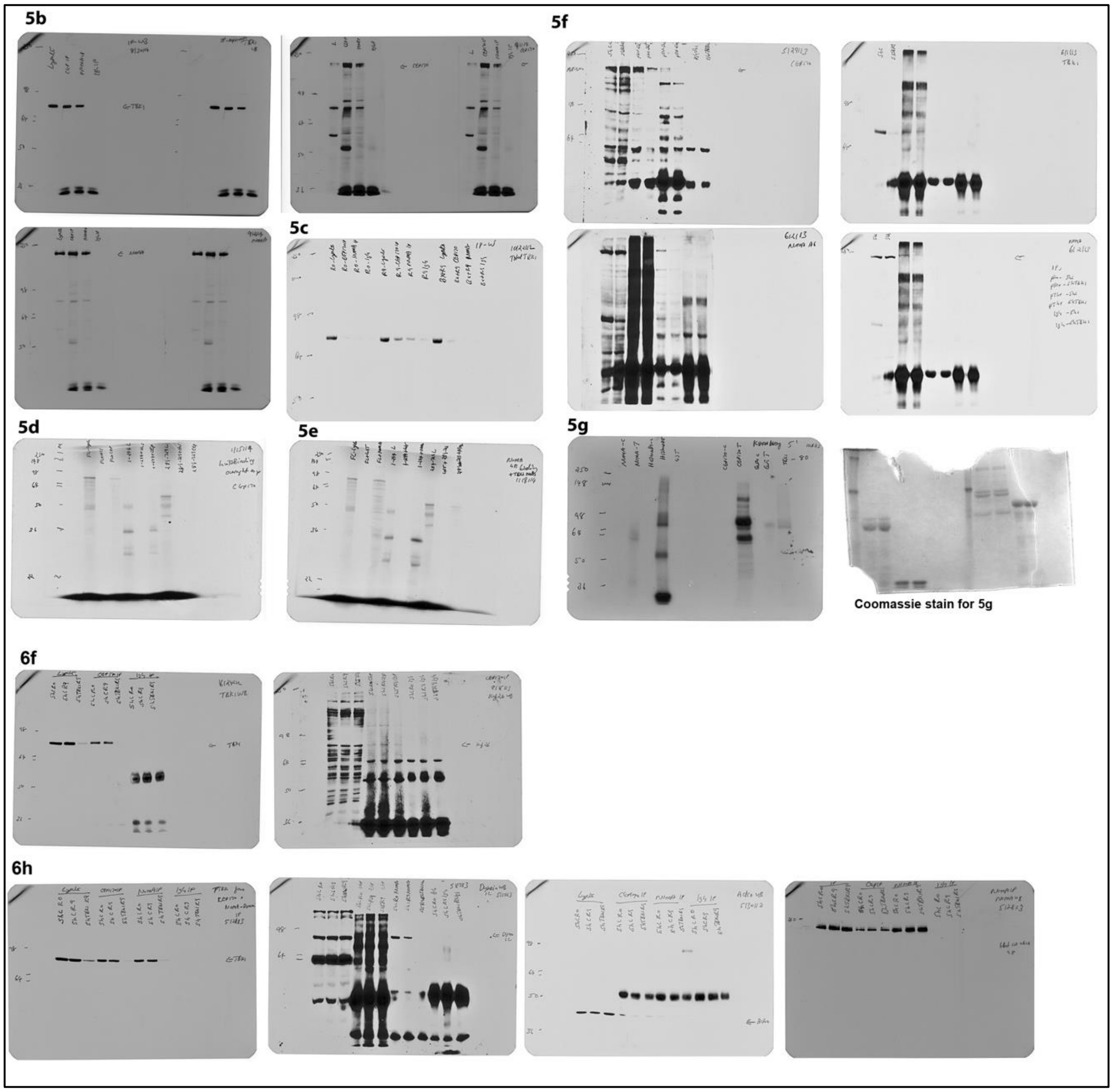
5b
5c
5d
5e
5f
5g
Coomassie stain for 5g
6f
6h

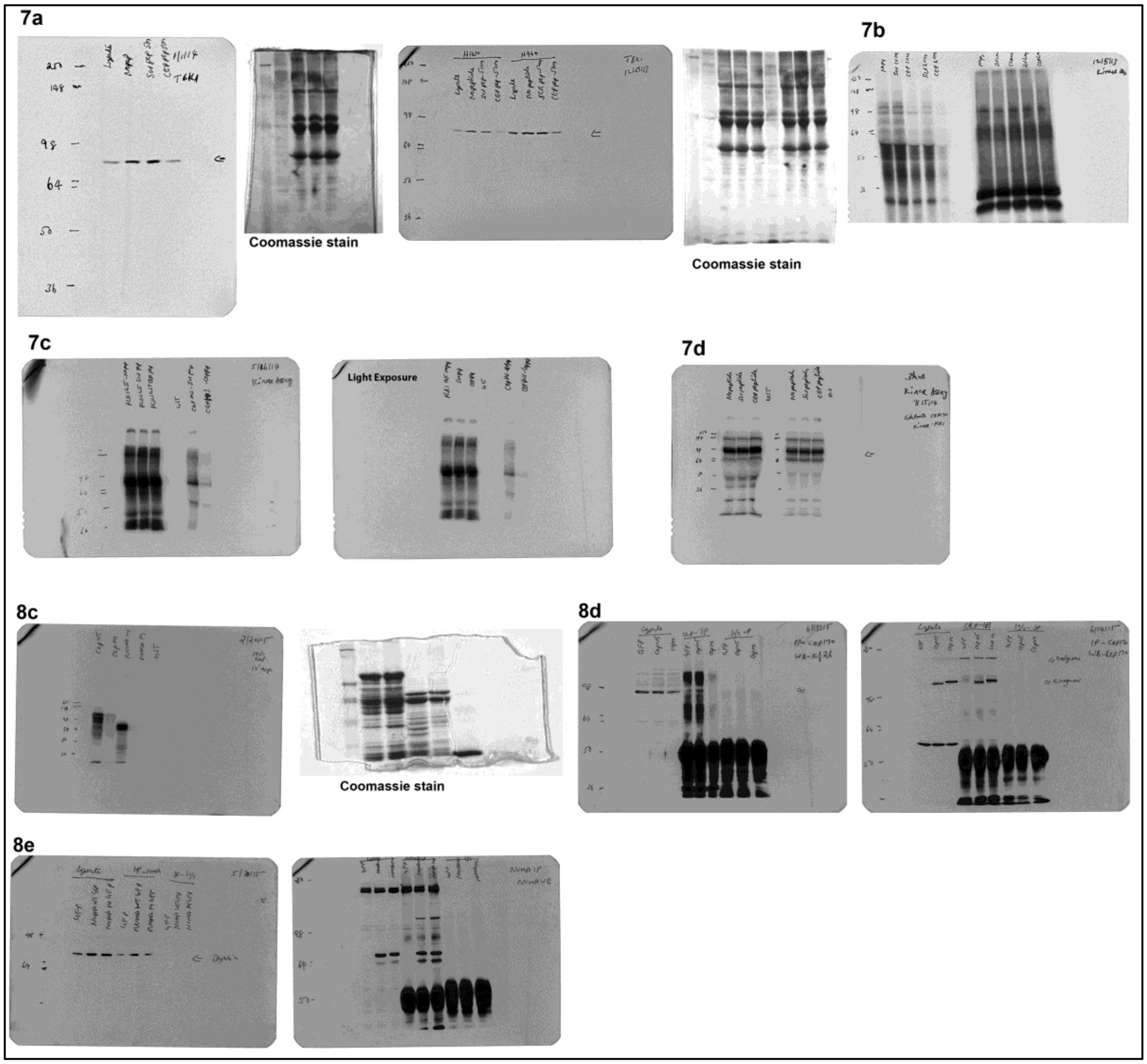
7a
Coomassie stain
Coomassie stain
7b
7c
Light Exposure
7d
8c
Coomassie stain
8d
8e

Supplementary Table 1

Peptide	Probability (%)	Start-Stop
**QTSSTPSSLALTSASR**	**95**	**859-874**
**ERSESLDPDSSMDTTLILK**	**95**	**877-895**
**DNSISPESDVDTASTISLVTGETER**	**95**	**926-950**
**LGEASDSELADADKASVASEVSTTS**	**95**	**1108-1141**
**LQSAGSAMPTSSSFK**	**95**	**1268-1282**
**VFDESLNFR**	**95**	**1358-1366**
**ALHPAAVSAAAEFENAESEADFSIHFNR**	**95**	**1543-1570**

Phosphorylated residues on CEP170 identified by mass spectrometry.

Phosphorylated Serine is indicated as 'S'
Phosphorylated Threonine is indicated as 'T'

For identifying CEP170 amino acid residues phosphorylated by TBK1, *in vitro* kinase assays were performed with purified His tagged CEP170 in the presence of TBK1. *In vitro* kinase reaction without TBK1 was performed as the negative control and the residues phosphorylated specifically in the presence of TBK1 were identified by mass spectrometry.

Supplementary Table 2

Peptide	Probability (%)	Start-Stop
EPQAKPQLDLSIDSLDLSCEEGTPLSITSK	100	1711-1740
LPPKVESLESLYFTPIPAR	100	1763-1781
LGSPDYGNSALLSLPGYRPTTR	95	1860-1881
RASMQPIQIAEGTGITTR	100	1967-1984
KLGNSLLR	99	2058-2065

Phosphorylated residues on NuMA identified by mass spectrometry

For identifying NuMA amino acid residues phosphorylated by TBK1, *in vitro* kinase assays were performed with purified GST tagged NuMA in the presence of TBK1. *In vitro* kinase reaction without TBK1 was performed as the negative control and the residues phosphorylated specifically in the presence of TBK1 were identified by mass spectrometry. Phosphorylated Serine is indicated as 'S'
